# Variation in herbivore defense strategies among plant species differing in elevational distribution and the role of temperature in defense

**DOI:** 10.1111/nph.70872

**Published:** 2026-01-07

**Authors:** Thomas Dorey, Janisse Deluigi, Alessio Maccagni, Sergio Rasmann, Gaétan Glauser, Yvonne Willi

**Affiliations:** ^1^ Plant Ecology and Evolution, Department of Environmental Sciences University of Basel 4056 Basel Switzerland; ^2^ Functional Ecology, Institute of Biology University of Neuchâtel 2000 Neuchâtel Switzerland; ^3^ Neuchâtel Platform of Analytical Chemistry, Faculty of Science University of Neuchâtel 2000 Neuchâtel Switzerland

**Keywords:** Brassicaceae, chemo‐diversity, constitutive and induced defenses, countergradient variation, elevational gradient, herbaceous plants, insect herbivory, temperature dependence

## Abstract

- Temperature influences the distribution and performance of both plants and insect herbivores. Consequently, plant–herbivore interactions are likely to vary across thermal gradients, which could affect the evolution of plant defense. Furthermore, temperature fluctuations may elicit immediate changes in defense.
- To study the evolutionary and ecological aspects of plant antiherbivore defense depending on temperature, we conducted a transplant experiment on a mountain slope involving 30 Brassicaceae species varying in elevational distribution. Additionally, we carried out a climate‐chamber experiment on a subset of 12 species to assess the temperature dependence of constitutive and induced defenses.
- The transplant experiment revealed that species from higher elevations experienced less herbivory than those from lower elevations. The climate‐chamber experiment demonstrated that high‐elevation species mounted stronger induced defenses in physical properties of leaves and in phytochemical diversity. Plant responses to low temperature, compared to control temperature, were lower constitutive defense and increased defense induction limited to leaf toughness. By contrast, high temperature increased constitutive chemical defense and defense‐induced leaf toughness.
- Results suggest higher herbivory resistance in high‐elevation Brassicaceae species by the induced remodeling of chemical defense. Such defense indication may have been shaped by rare but hard‐to‐tolerate herbivory in the evolutionary past.

## Introduction

The spatial distribution of species is influenced by numerous ecological factors, with climate ranking among the most important (Gaston, 2003). Indeed, climate can explain the current latitudinal and elevational distribution of many species (Lee‐Yaw *et al*., 2016; Paquette & Hargreaves, 2021; Patsiou *et al*., 2021), producing turnover in communities over these geographic scales. Concomitantly, turnover in species interactions is observed along latitudinal and elevational gradients, such as those between plants and their herbivores (Novotny & Weiblen, 2005; Pellissier *et al*., 2012; Hargreaves *et al*., 2019). Yet, while climate can influence which plants and herbivores co‐occur across space, it can also affect further aspects of plant–herbivore interactions, such as by altering plant growth or plant defense (Maron *et al*., 2014; Hamann *et al*., 2021). Therefore, predicting the geographical mosaic of plant–herbivore interactions requires an understanding of how plant traits that mediate these interactions vary in response to climate, through evolved and immediate responses (Merrill *et al*., 2008; Adams & Zhang, 2009; Dostálek *et al*., 2016).

The majority of terrestrial herbivores – both in abundance and diversity – are insects (Huntly, 1991). As ectotherms, their physiology, development, and lifetime performance are intrinsically linked to temperature (Bale *et al*., 2002). Accordingly, plant–insect herbivore interactions are predicted to become less important toward cooler climates, toward the poles (Schemske *et al*., 2009; Anstett *et al*., 2016), or at higher elevation (Körner, 2007; Rasmann *et al*., 2014b). Although several studies have found a decrease in the density and species richness of herbivores at higher latitudes and elevations (Pennings *et al*., 2009; Salazar & Marquis, 2012; Moreira *et al*., 2018), others have reported contradictory findings within the same study region or along different elevational gradients (Merrill *et al*., 2008; Adams & Zhang, 2009; Dostálek *et al*., 2016). These results highlight additional factors impacting the abundance of plant–insect herbivore interactions over these gradients, such as differences in plant defense deployment (Johnson & Rasmann, 2011).

In order to defend themselves against herbivores, plants have evolved a variety of strategies. These range from mechanisms that enable them to compensate for tissue loss (Agrawal *et al*., 1999) to traits that deter herbivores from settling, feeding, and reproducing, or that reduce palatability (Karban *et al*., 1997; Mitchell *et al*., 2016). However, defense incurs costs as it typically involves diverting resources away from growth and reproduction, resulting in a growth–defense trade‐off (Strauss *et al*., 2002). Therefore, defense traits are often expressed not constitutively but plastically (i.e. induced) in response to herbivores (Karban, 2011; Moreira *et al*., 2014). Induced defense may be less demanding of resources and favored when costs arise only upon activation and when attacks are rare; however, it can be risky if a delayed response fails to prevent severe damage (Karban, 2011). Constitutive and induced defenses are predicted to covary antagonistically, within and across species (Agrawal *et al*., 2010) as well as along ecological/climatic gradients (DeLucia *et al*., 2012; Pellissier *et al*., 2012). For example, along elevational gradients, constitutive defense is predicted to be higher at low elevations, where herbivore pressure is strong and persistent, while induced defense should be higher at high elevations, where herbivory is sparse (Rasmann *et al*., 2014a).

Beyond this distinction between constitutive and induced defense, a significant number of studies have reported increased defense levels at higher elevations (reviewed by Moreira *et al*., 2018). Higher defense in plants of high elevations may be an adaptation to avoid tissue loss, which may be more difficult to compensate for in high‐elevation habitats with a shorter growing season. Alternatively, improved defense may be a by‐product of adaptation to cope with high‐elevation climatic conditions, which indirectly increases plant resistance to herbivores (Rasmann *et al*., 2014b; Abdala‐Roberts *et al*., 2016; Pellissier *et al*., 2016). Plants living in alpine environments often have a higher leaf dry matter content and thicker leaves than their low‐elevation counterparts (Körner, 2003; Scheepens *et al*., 2010, but see Maccagni & Willi, 2022 for Brassicaceae). These traits favor leaf sturdiness and longevity, and such differences in leaf structure might also influence plant–herbivore interactions (Kergunteuil *et al*., 2018). For instance, Guerra *et al*. (2010) demonstrated that thicker leaves in response to abiotic factors reduced palatability and herbivore performance. Thus, a better understanding of leaf structural changes along elevational gradients and their implications in plant–herbivore interactions is needed.

In addition to evolved functional‐trait patterns, temperature fluctuations throughout a plant's lifetime can influence its defensive strategies. Under elevated temperatures, plants may enhance defense in a plastic manner, for example by upregulating the specialized metabolome to deter herbivores (Bale *et al*., 2002; Jamieson *et al*., 2012; Wang *et al*., 2020b). However, increased secondary metabolite production is not exclusive to warmer temperatures. Similar trends of increased glucosinolate levels have been observed in *Brassica oleracea* L. and *Arabidopsis thaliana* (L.) Heynh. when grown at low temperatures (Pereira *et al*., 2002; Kissen *et al*., 2016). Nevertheless, other studies have demonstrated that plant species cultivated in cold environments exhibit reduced constitutive defenses (Pellissier *et al*., 2016; Buckley *et al*., 2023). Together, these findings emphasize the complex relationship between temperature and plant defense, adding to the difficulty of understanding the mechanisms that drive the dynamics of plant–herbivore interactions along latitudinal and elevational gradients.

The aim of this study was to investigate differences in constitutive and induced defenses among species with different elevational distributions (evolved differences) and plastic temperature responses in defense (immediate differences). We selected 30 Brassicaceae species with low‐ to high‐elevation distributions in Central Europe and raised them in a transplant experiment across five sites on a mountain slope ranging from 600 to 2000 m above sea level (m asl). We estimated herbivore diversity and abundance, as well as damage to the plants. In a climate‐chamber experiment involving three thermal regimes (cold, benign, and warm) and a subset of 12 species, we assessed constitutive and induced defenses in leaf structural traits and glucosinolate production. Based on the reasoning outlined above, we tested a suite of hypotheses: (1) Herbivore abundance and damage decrease with increasing elevation of transplant sites. (2) Low‐ and high‐elevation species are equally preyed on by herbivores in the transplant experiment. (3) Low‐elevation species achieve protection by higher constitutive defense, whereas high‐elevation species express higher herbivory‐induced defense (evolved differences; in climate‐chamber experiment). (4) Due to an allocation trade‐off, species with increased defense have slower growth and are smaller. (5) Cooler temperature lowers the expression of defense traits, and higher temperature increases it (immediate differences in response to temperature across species; in climate‐chamber experiment). The results contribute to a deeper understanding of how temperature mediates plant–herbivore interactions on evolutionary and ecological time scales.

## Materials and Methods

### Plant species and sampling

The Brassicaceae family comprises *c*. 310 genera and 3500 species distributed worldwide (Al‐Shehbaz, 2011). The family is characterized by the production of specific secondary metabolites known as glucosinolates, which are anionic thioglucosides (Halkier & Gershenzon, 2006). These metabolites act as deterrents for insect herbivores when activated by myrosinases (Hopkins *et al*., 2009). For the transplant experiment (as mentioned in the later section), we selected and collected seeds from 30 species of the Brassicaceae family (Supporting Information Methods S1; Table S1) that had similar edaphic requirements (moisture indicator values between 2 and 4; Landolt *et al*., 2010) and represented low‐ and high‐elevation taxa across the European Alps. For the climate‐chamber experiment, we selected 12 species from the original 30 based on the following criteria: (1) equal representation of low‐ and high‐elevation species; and (2) good representation of the phylogeny (avoiding overrepresentation of one genus).

### Field transplant experiment

#### Sites

From fall 2018 to summer 2019, we conducted a transplant garden experiment in the Swiss Alps (Calanda mountain, 46.875°N, 9.501°E, Switzerland). Along an elevational gradient spanning over a distance of 3 km, we selected five sites at 601, 997, 1395, 1610, and 1998 m asl. The sites were on an east‐facing mountain slope consisting of calcareous bedrock. The climate gradient was continuous, with the mean annual temperature (5 cm above the ground) ranging from 10.20°C at the lowest site to 4.08°C at the highest site (Table S2; Fig. S1A). However, the mean temperature during leaf herbivory assessments in fall and spring, defined by the onset and end of winter conditions, respectively, did not show a consistent decline with elevation (Fig. S1B,C).

#### Transplantation

Transplantation started at the highest site and ended at the lowest site 2 months later (Table S2). Transplanting to each site was done once the long‐term daily mean temperature dropped to *c*. 10°C (source of information: www.meteoschweiz.admin.ch). Before transplanting, the seeds were sown in climate chambers, where the seedlings were kept for *c*. 1 month (see Methods S2). At each site, 600 plants were distributed in two spatial blocks arranged in parallel rows of multipot trays (30 species × 2 populations × 5 families × 2 replicates/blocks, for a total of 3000 plants across the five sites). The trays were buried in the soil up to their rims. To protect the transplant sites from large herbivores, a 1‐m‐high fence was installed (Fig. S2). To standardize soil conditions and ensure that the main factors varying across sites were climate and insect herbivore pressure, we used pots filled with the same substrate across all transplant sites.

At each site, we recorded the aboveground temperature (5 cm above the ground; ‘micro‐climatic temperature’) every 15 min using four climate loggers per site (thermologger; TOMST s.r.o., Prague, Czech Republic) set randomly above empty pots and distributed across the two blocks.

### Climate‐chamber experiment

#### Experimental design

The 12 species (Table S1) were grown under experimental conditions involving manipulated temperature regimes (cold, benign, and warm) and defense induction (with or without methyl jasmonate [MeJA]) in a fully factorial design. The latter treatment was used to simulate the plant response to chewing herbivores. MeJA is a plant hormone with many functions (Cheong & Do Choi, 2003); one of them is activating plant defense in response to insect‐driven wounding (McConn *et al*., 1997). The downstream effect is the production of proteins that are toxic to insect herbivores (e.g. Zhang *et al*., 2015). The experimental application of MeJA to induce defense is preferred to wounding (e.g. cutting) because a more specific response is achieved (McConn *et al*., 1997; Reymond *et al*., 2000). We randomly selected two families of seeds per population and species (or four seed families when only one population was sampled) and sowed seeds of each family in six pots, one for each treatment combination (12 species × 2 populations × 2 families × 3 temperatures × 2 herbivory induction treatments = 288 plants). The sowing and early plant rearing were similar to those in the transplant study (Methods S2), except for a few minor differences: Sowing took place in spring 2019, and the seeds were sown in single square pots measuring 7 × 7 × 8 cm (0.4 l) with holes at the bottom (Desch 5°; gvz‐rossat.ch, Oetelfingen, Switzerland).

#### Treatments

Once the plants had reached the two‐leaf stage, they were randomly assigned to one of the six treatment combinations in separate growth chambers (CLIMECAB; Kälte 3000, Landquart, Switzerland) to prevent cross‐contamination from herbivory‐induced volatile compounds. The temperature treatments were as follows: cold (constant 14°C), benign (constant 20°C), and warm (constant 27°C). These temperatures encompassed the range that could be experienced when moving from the colline to the alpine zone during summer days (Justen & Fritz, 2013). Light (12 h of white LED light at 380 μmol m^−2^ s^−1^) and relative humidity (50%) were kept constant in the six chambers. Then, the plants in half of the chambers were treated with the exogenous application of MeJA. Open leaves of individual plants were treated once a week for 6 wk with 250 μl of MeJA solution (4.5 μl 0.005% Triton X‐100 (CAS Number 9002‐93‐1; Sigma‐Aldrich), 220 μl ethanol (≥ 99.8%), 43.75 ml deionized water, 80 μl of 9.5 μM MeJA (CAS Number 77026‐92‐7; Sigma‐Aldrich)). The other half of the plants served as controls and received the same treatment, except MeJA was replaced with an equivalent volume of deionized water. We randomized pot positions in each growth chamber once a week. Once the plants started slowing growth, the treatments were stopped, and leaf samples were collected for phenotyping and chemical analysis.

### Trait assessment

#### Herbivore abundance in the transplant experiment

To evaluate herbivore abundance along the elevational gradient, we used sweep net sampling and pitfall traps. In spring/summer 2019, we performed sweep net sampling twice at each site and installed five pitfall traps per site that were checked weekly for 4 wk (Methods S3; Table S2). We identified insects to the level of the order and only retained plant feeders described as such in the literature (Speight *et al*., 1999) for analyses.

#### Herbivore damage in the transplant experiment

During fall 2018 and spring 2019, we assessed leaf herbivore damage on the transplanted plants weekly for 8 wk. We defined fall as the period between transplantation and the day when the daily mean temperature changed to ≤ 0°C for 6 consecutive days. Spring began when the mean daily temperature was > 0°C for 6 consecutive days. Once spring started, we waited 5–6 wk to allow for plant recovery and insect emergence after winter (Table S2 with details on start dates). We scored herbivory on a scale from 0 to 5: 0 for no damage, 1 for small feeding marks on the leaves (< 5% of leaves damaged), 2 for ≤ 25% of the leaves partially or entirely eaten, 3 for ≥ 25% of the leaves partially or entirely eaten, 4 for ≥ 75% of the leaves partially or entirely eaten, and 5 for a totally eaten plant. Since damage levels measured weekly throughout the season were highly correlated with final damage levels (as only a few plants compensated for damage), we used end‐of‐season damage levels for further analyses. Plants started the seasons mostly undamaged, and early‐spring damage levels (week 1) were uncorrelated with damage observed in fall.

##### Plant growth in the climate‐chamber experiment

We estimated plant growth by measuring the length of the two longest leaves twice a week for the first 2 wk after treatment start and once a week for the subsequent 7 wk. Length data were used to fit seven growth models (linear, exponential, power, 2‐ and 3‐parameter logistic, Gompertz, and von Bertalanffy). On average, the 3‐parameter logistic model produced the best fit, as determined by the weighted AIC for each model per plant (Methods S4; Table S3; Fig. S3). We extracted the following parameters from this model: time to reach half the asymptotic size (x_mid_, in days), growth rate (the inverse of the scale factor), and asymptotic size (in cm).

##### Plant physical defense in the climate‐chamber experiment

When the plants were about to reach their asymptotic size, we sampled two leaves of the same developmental stage from each plant. We used specific leaf area (SLA, mm^2^ mg^−1^), which is the ratio of leaf area to dry mass, and leaf dry matter content (LDMC, mg g^−1^), which depicts the ratio of leaf dry weight to fresh weight, as proxies for structural defense and digestibility. Lower SLA and higher LDMC indicate increased structural defense and reduced digestibility (Hanley & Sykes, 2009). Although these two variables tend to be inversely correlated, both are typically included in studies of structural defenses against herbivory. Immediately after harvest, we measured the fresh weight of each leaf with an analytical balance (AT250, XA205 DualRange, precision 0.01 mg; Mettler Toledo, Columbus, OH, USA), scanned the leaf (CanonScan, LiDe120; Canon, Tokyo, Japan), and estimated its surface area with the software ImageJ (Abràmoff *et al*., 2004). The dry weight of each leaf was measured after 48 h at 60°C.

##### Plant chemical defense in the climate‐chamber experiment

To measure glucosinolate content, we sampled *c*. 50 mg of fresh leaf tissue per plant. The tissue was flash frozen in liquid nitrogen, stored at −80°C, and later lyophilized for 48 h (CoolSafe Touch 95–15, at −95°C; LaboGene, Allerød, Denmark). Leaf glucosinolate analysis was performed at the Neuchâtel Platform for Analytical Chemistry (NPAC, University of Neuchâtel, Switzerland). Five milligrams of dry matter (±1 mg) per sample were ground into a powder using a tissue lyser (Mixer Mill MM400; Retsch, Haan, Germany) with two 3‐mm‐thick stainless‐steel beads for 2 min at 30 Hz, and 1 ml of ice‐cold methanol (MeOH) dissolved in water with formic acid (70 : 30 : 0.1; methanol HPLC grade) per sample was added. The samples were vortexed for 10 s and shaken in a tissue lyser (Mixer Mill MM400; Retsch) for 4 min at 30 Hz. Then, the samples were centrifuged for 4 min at 14 000 ***g***, and the supernatant was diluted 20‐fold with the extraction solution. The samples were analyzed by ultra‐high‐performance liquid chromatography–high‐resolution mass spectrometry (UHPLC–MS), as described by Glauser *et al*. (2012). We identified 57 different candidate glucosinolate compounds. Indole glucosinolates were quantified as glucobrassicin equivalents while others (e.g. aliphatic, benzoyl) were quantified as glucoraphanin equivalents.

Total glucosinolate production (μg g^−1^) was the sum of the concentrations of all detected compounds. The total concentration of aliphatic, aromatic, and indole glucosinolates was calculated by summing the concentrations of compounds in these three classes (Fahey *et al*., 2001). Furthermore, we calculated three phytochemical diversity indices: one emphasizing richness – the number of compounds (glucosinolate richness); one emphasizing evenness – Simpson's diversity index; and one emphasizing functional aspects – Rao's quadratic entropy (RaoQ), using the chemodiv package (Petrén *et al*., 2023). For the latter index, we considered chemical class (aliphatic, aromatic, indole), the class of breakdown (isothiocyanate, oxazolidine‐2‐thione, and indol‐3‐carbinol), and the molecular weight as functional traits of each glucosinolate, as suggested in Bakhtiari *et al*. (2021).

### Statistical analysis

All analyses and figures were performed using R v. 4.3.2 (R Foundation for Statistical Computing, Vienna, Austria, 2023). Statistical analyses were based on Bayesian generalized linear mixed models (brms package; Bürkner, 2017). Posteriors are reported as median values, and significance was evaluated based on 90% credible intervals (CI90) of high‐density intervals (HDI) and the probability of direction using the bayestestR package (Makowski *et al*., 2019). Values were drawn from four independent, parallel chains. Burn‐in, number of iterations, thinning interval, maximal tree depth, and adaptive delta were adjusted for each model to ensure an effective sampling size (ESS) of at least 1000. Model convergence was checked by inspecting diagnostic plots visually and Rhat values. Before analyses, median elevation of species' occurrences (in m asl) in Switzerland (based on data from infoflora.ch; Patsiou *et al*., 2021) was log‐transformed, and continuous explanatory variables were mean‐centered. Where applicable, relatedness among species was included as a random effect. The phylogeny, including all studied species and more (Patsiou *et al*., 2021), was pruned using the ‘treedata’ function of the *geiger* package (Harmon *et al*., 2008). The relatedness matrix was generated using the ‘vcv’ function in the ape package (Paradis & Schliep, 2019) and called with the ‘cov_ranef’ parameter of brm. For all analyses, we assessed the contribution of phylogeny by comparing model fits with and without phylogeny using the leave‐one‐out (LOO) cross‐validation criterion. We considered a difference in LOOIC to indicate a significant effect of phylogeny if the estimated difference exceeded its SE (i.e. elpd_diff/se_diff > 1; Vehtari *et al*., 2017).

#### Herbivore abundance on the elevational gradient (H1)

We tested for an association between herbivore abundance and elevation. Fixed effects were elevation of transplant sites, sampling method, and their interaction. Random effect was site. The dependent variable was herbivore count summed over the daily sweep net attempts at each site, or the 5 pitfall traps per site and week (*N* = 30). Data were log‐transformed before analyses to approach normality of the residuals. Additionally, we used a model with mean annual temperature at transplant sites instead of elevation to test whether herbivore abundance was influenced more directly by local temperature (i.e. microclimate). Mean annual temperature at transplant sites was calculated for the period from 9 October 2018 to 21 August 2019, representing the common time frame over which temperature was recorded at all transplant sites.

#### Herbivore damage on the elevational gradient (H1, H2)

We analyzed leaf damage by herbivores on the transplanted species using elevation of transplant sites, median elevation of species' occurrences (Patsiou *et al*., 2021), and their interaction as fixed effects. Random effects were site, block nested within site, species, population nested within species, family nested within population and species, and relatedness among species. The dependent variable, total leaf damage, was modeled assuming an ordinal distribution (*family* = cumulative). Separate analyses were conducted for fall and spring. For the spring time, the analysis was repeated by replacing the elevation of the sites with the average herbivore abundances revealed by sweep net sampling and in pitfall traps. Finally, we ran an additional type of model in which elevation of transplant sites was replaced by mean annual temperature during the course of the experiment at each transplant site.

##### Defense and plant growth in the climate‐chamber experiment (H3–H5)

The analyzed variables included physical defense traits (i.e. SLA and LDMC), chemical defense variables (i.e. glucosinolate concentrations and chemical diversity), and growth traits (i.e. x_mid_, growth rate, asymptotic size). For analysis, all growth and physical defense traits were transformed using log_10_(*x* + 1). Since glucosinolate production was zero‐inflated, with a significant number of plants producing no glucosinolates (52 out of 250 individuals), we modeled zero‐inflated gamma distributions. Diversity indices were analyzed using beta distributions (after standardization of *x*_*i*_/(max(*x*) + 1)). Fixed effects were median elevation of species' occurrences, herbivory/MeJA induction, growth temperature, and the interaction between herbivory induction and temperature; LOO cross‐validation had revealed that the three‐way interaction and the two‐way interaction involving elevation of species' occurrences and growth temperature did not greatly improve model performance (Table S4). Random effects were species, population nested in species, family nested in population and species, and relatedness among species. Contrasts were performed by comparing each treatment against the reference level: ‘noninduced’ for herbivory/MeJA induction and ‘benign’ for temperature.

## Results

### Field transplant experiment

#### Herbivore abundance on the elevational gradient (H1)

**Fig. 1:**
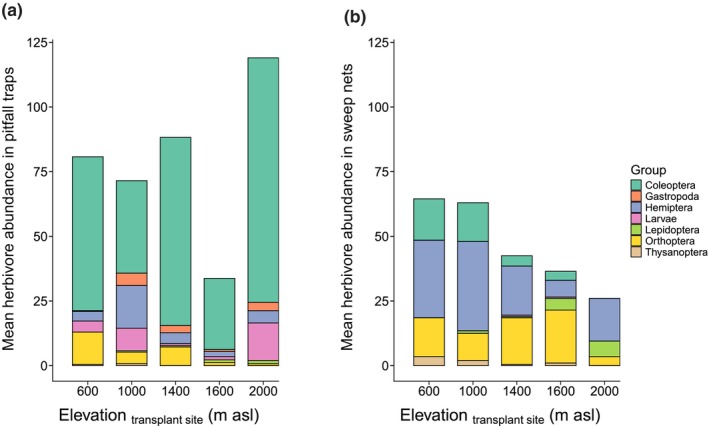
Herbivore abundance and diversity in pitfall traps (a) and sweep nets (b) across the elevational gradient in the field transplant experiment. Mean herbivore abundance is expressed as the average number of individuals collected per sampling event at each transplant site. The representation of insect orders or other taxonomic groups and of larvae is indicated in colors.

The two sampling methods produced significantly different herbivore counts, with higher numbers in pitfall traps that were out for 1 wk before being emptied (Table S5; Fig. 1). Most of the insects sampled were from the order Coleoptera, followed by Hemiptera, Orthoptera, Lepidoptera, and Thysanoptera, as well as unidentified larvae of Coleoptera and/or Lepidoptera (Fig. 1). A further group of herbivores were land snails (Gastropoda). When combining counts from both sampling methods, we found the highest herbivore abundance at the lowest site (600 m asl) and the lowest at the second‐highest site (1600 m asl). However, the association with elevation was not significant in the overall model (Table S5; Fig. S4A–D). Yet, we found a significant interaction between elevation and sampling method, with insect numbers caught by sweep net sampling declining with increasing elevation, but not by pitfall traps (Table S5; Fig. 1). When elevation was replaced by mean annual temperature measured at the transplant sites, the effect of sampling method remained, but the interaction was not significant (Table S5). Overall, there was an indication of reduced abundance of some herbivores at higher elevations.

#### Herbivore damage on the elevational gradient (H1 and H2)

**Fig. 2:**
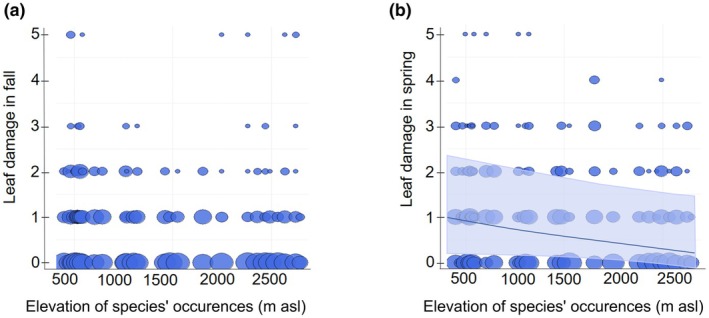
Leaf herbivore damage varying with elevation of species' occurrences in fall (a) and in spring (b) in the field transplant experiment. The size of circles represents the number of plants of a species with a particular damage level. The line in (b) represents the model‐predicted regression, while the shaded area indicates the 95% confidence interval around the model prediction.

Despite this indication of varying herbivore abundance over elevation, herbivore damage in the first fall and in the following spring did not significantly change with the elevation of transplant sites, nor was there a significant interaction between elevation of sites and median elevation of species' occurrences (Table S6). Also, herbivore damage in spring was not significantly associated with the two estimates of herbivore abundance in sweep nets and in pitfall traps (Table S6). In this second model though, the interaction between abundance of herbivores in sweep nets and elevation of species' occurrences was significant, with the result being hard to interpret as with increasing insect numbers, less damage was observed (Fig. S4D). Finally, when mean annual temperature (MAT) was used to replace elevation of transplant sites in a third type of model, neither MAT nor its interaction with elevation of species' occurrences was significant. Herbivore damage was not lower at higher elevations or in cooler climates. However, high‐ compared to low‐elevation plants experienced less damage by herbivores in the spring survey (Fig. 2b). This result was revealed by all three types of models. A comparable but nonsignificant trend was apparent in fall (Fig. 2a; Table S6). In these and all further analyses, species relatedness did not explain significant amounts of variation.

### Climate‐chamber experiment

#### Defense and plant growth (H3, H4)

Under benign temperature, species from low and high elevations did not differ in constitutive defense (Table 1, means of treatment combinations in Table S7). The only traits to differ with the median elevation of species' occurrences were maximal growth rate and asymptotic size. High‐elevation species grew significantly slower and to smaller size than low‐elevation species. Correlation analysis on estimates under benign temperature and across species revealed only three significant associations between growth and defense traits (Table S8, lower left): higher values of LDMC were associated with shorter time to half size, higher maximal growth rate, and higher asymptotic size. Results suggested no evolved difference in constitutive defense among species over the elevational gradient and no detectable trade‐off between growth and defense among control plants.

**Table 1:** Effects of median elevation of species' occurrences, herbivory induction, temperature, and interactions on defense and growth traits estimated by mixed‐effects models (climate‐chamber experiment).

Traits	*n*	$R_{c}^{2}$	Elevation of species (ESpecies)	Herbivory induction (H)	H × ESpecies	Temperature (*T*)		H × T	
					Slope on E of MeJA‐treated vs nontreated plants	Cold vs benign	Warm vs benign	Cold vs benign	Warm vs benign
Defense traits									
Leaf dry matter content	248	0.75	−0.13 [−0.42, 0.15]	0.03 [0.00, 0.06]	0.11 [0.05, 0.18]***	−0.05 [−0.08, −0.03]***	−0.09 [−0.12, −0.07]***	0.09 [0.06, 0.13]***	0.06 [0.02, 0.10]**
Specific leaf area	247	0.68	0.20 [−0.14, 0.50]	−0.05 [−0.08, −0.01]*	−0.17 [−0.25, −0.10]***	0.03 [0.00, 0.06]	0.11 [0.07, 0.14]***	−0.08 [−0.13, −0.04]***	−0.02 [−0.07, 0.03]
Conc. total glucosin.									
Logistic^1^	250	0.60	−9.61 [−21.77, 1.51]	−2.23 [−4.28, −0.42]*	4.56 [−0.13, 9.47]	−0.31 [−1.91, 1.29]	−4.72 [−7.88, −2.11]***	3.12 [0.76, 5.66]*	0.93 [−3.56, 4.96]
Gamma			0.23 [−1.06, 1.65]	0.53 [0.02, 1.03]	−0.24 [−1.26, 0.75]	−0.67 [−1.22, −0.18]*	0.59 [0.08, 1.07]*	−0.11 [−0.85, 0.58]	−0.23 [−0.96, 0.52]
Conc. aliphatic glucosin.									
Logistic^1^	250	0.61	−9.85 [−22.87, 1.59]	−1.65 [−3.64, 0.12]	3.65 [−1.08, 8.65]	−0.35 [−1.96, 1.29]	−4.82 [−8.11, −2.23]***	2.53 [0.18, 5.11]	0.33 [−4.10, 4.35]
Gamma			0.48 [−0.93, 1.95]	0.44 [−0.10, 0.91]	−0.11 [−1.13, 1.01]	−0.77 [−1.28, −0.17]*	0.60 [0.12, 1.12]*	−0.07 [−0.76, 0.70]	−0.15 [−0.86, 0.63]
Conc. aromatic glucosin.									
Logistic^1^	250	0.31	−1.33 [−9.16, 6.35]	−2.36 [−3.76, −0.99]**	−3.29 [−6.80, −0.01]	2.79 [1.33, 4.47]**	−2.71 [−4.11, −1.40]***	0.66 [−1.46, 2.80]	1.94 [−0.25, 4.20]
Gamma			−0.04 [−1.55, 1.53]	−0.01 [−0.35, 0.35]	−0.32 [−1.06, 0.46]	−0.00 [−0.50, 0.50]	0.25 [−0.09, 0.58]	0.11 [−0.52, 0.73]	−0.10 [−0.60, 0.37]
Conc. indole glucosin.									
Logistic^1^	250	0.29	−1.69 [−8.81, 5.91]	−1.88 [−3.11, −0.72]**	0.47 [−2.54, 3.55]	2.41 [1.02, 4.04]**	−2.59 [−3.98, −1.35]***	0.47 [−1.50, 2.29]	2.85 [1.01, 4.79]***
Gamma			−0.06 [−1.28, 1.20]	−0.38 [−0.78, 0.05]	−0.58 [−1.49, 0.23]	−0.11 [−0.66, 0.44]	−0.18 [−0.58, 0.20]	0.70 [0.01, 1.38]	0.11 [−0.49, 0.67]
Glucosinolate richness	250	0.32	0.65 [−0.56, 1.83]	0.19 [−0.14, 0.54]	−0.11 [−0.85, 0.66]	−0.97 [−1.35, −0.64]***	0.63 [0.29, 0.99]**	0.16 [−0.34, 0.65]	−0.55 [−1.09, −0.05]
Simpson's diversity index	250	0.61	0.04 [−0.50, 0.66]	0.05 [−0.05,0.15]	0.33 [0.10, 0.54]*	−0.13 [−0.24, −0.04]*	−0.00 [−0.10, 0.09]	0.12 [−0.02, 0.26]	0.12 [−0.03, 0.26]
Rao's quadratic entropy	250	0.66	0.30 [−1.01, 1.61]	−0.06 [−0.36, 0.27]	0.43 [−0.24, 1.07]	−0.53 [−0.85, −0.21]**	0.05 [−0.25, 0.34]	0.34 [−0.10, 0.80]	−0.14 [−0.56, 0.28]
Growth traits									
Time to half size	244	0.36	0.10 [−0.01, 0.20]	−0.13 [−0.17, −0.09]***	−0.42 [−0.53, −0.32]***	0.03 [−0.01, 0.06]	−0.00 [−0.04, 0.04]	−0.06 [−0.12, −0.01](.)	−0.12 [−0.18, −0.05]***
Maximal growth rate	244	0.15	−0.02 [−0.04, −0.01]***	−0.01 [−0.01, −0.01]***	0.00 [−0.01, 0.01]	−0.01 [−0.01, −0.01]***	−0.00 [−0.01, 0.00]	0.01 [0.00, 0.00]	0.00 [0.00, 0.01]
Asymptotic size	284	0.75	−0.17 [−0.61, 0.30]	−0.29 [−0.39, −0.20]***	−0.26 [−0.30, −0.21]***	−0.11 [−0.15, −0.07]***	−0.01 [−0.05, 0.04]	0.00 [−0.06, 0.06]	−0.04 [−0.11, 0.02]

^1^ For logistic process: prediction of 0. Sample sizes (*n*), conditional *R* ^2^ ($R_{c}^{2}$) and coefficients of fixed effects, the median and 90% high‐density interval (HDI) of the posterior distribution are reported. Significant effects are indicated in bold (HDI not overlapping with 0 and probability of direction (pd) > 97.5% [(.), > 95%; *, > 97.5%; **, > 99.5%; ***, > 99.95%]). Results for random effects are not presented.

**Fig. 3:**
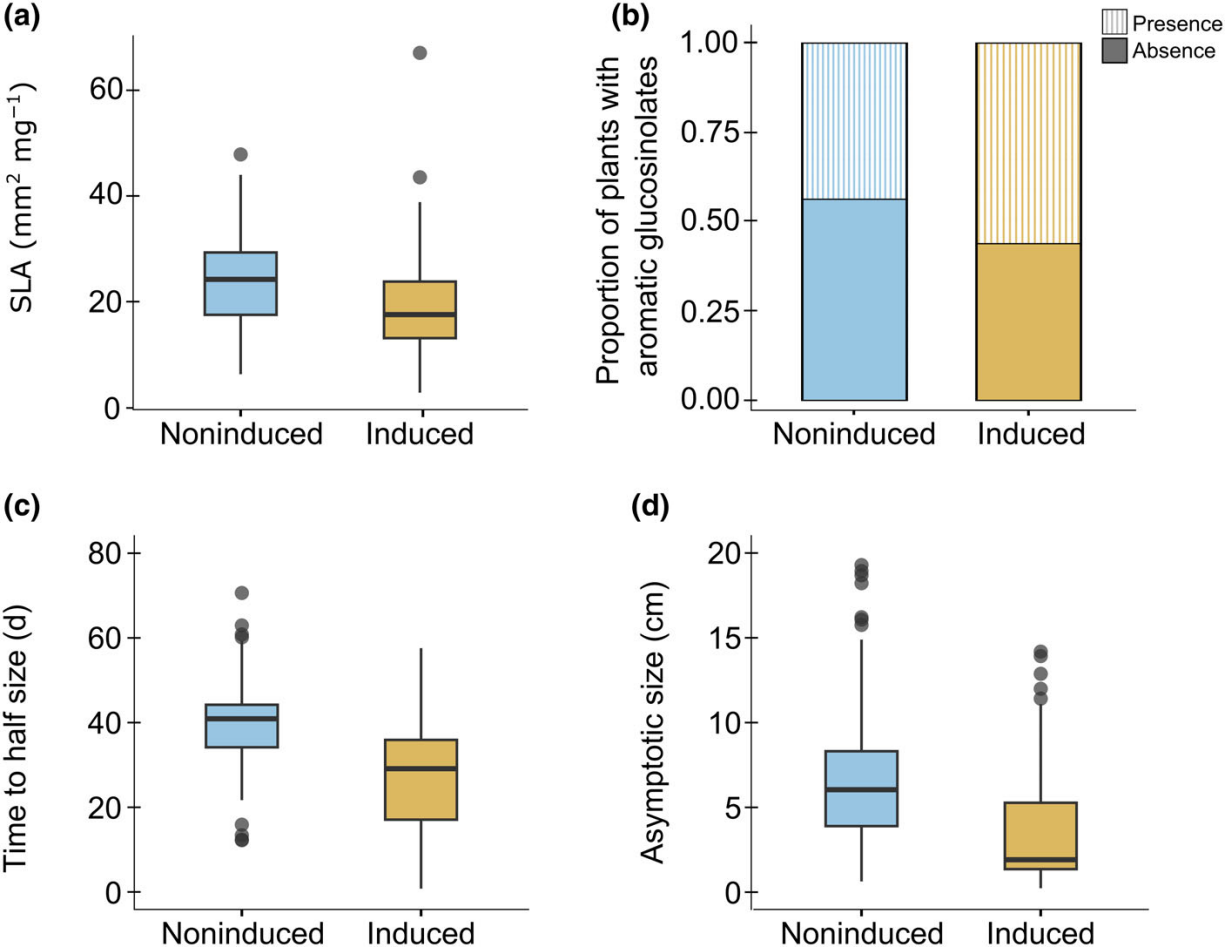
Distribution of defense and growth traits for noninduced and herbivory‐induced plants in the climate‐chamber experiment: (a) specific leaf area; (b) proportion of plants with aromatic glucosinolates; (c) time to half size; (d) asymptotic size. The thick line of boxplots represents the median across plants of the 12 Brassicaceae species, the box indicates the lower and upper quartiles, the whiskers extend to the smallest and largest values within 1.5 times the IQR from the quartiles, and points beyond this range are outliers (untransformed data). Herbivory induction was done with MeJA (blue: noninduced; brown: herbivory‐induced).

Treatment with MeJA, which simulates herbivore attack, led to an induced decrease in SLA and fewer plants with nondetectable glucosinolate concentrations, for total glucosinolates, aromatic glucosinolates, and indole glucosinolates (Table 1, Fig. 3a,b). Furthermore, MeJA treatment was associated with shorter time to half size, lower maximal growth rate, and smaller asymptotic size (Fig. 3c,d). Correlation analysis again revealed no significant evidence for a trade‐off between growth and defense in induced plants under benign temperature (Table S8, upper right). The only significant correlation was between Simpson's diversity in glucosinolates and maximal growth rate, and it was positive.

**Fig. 4:**
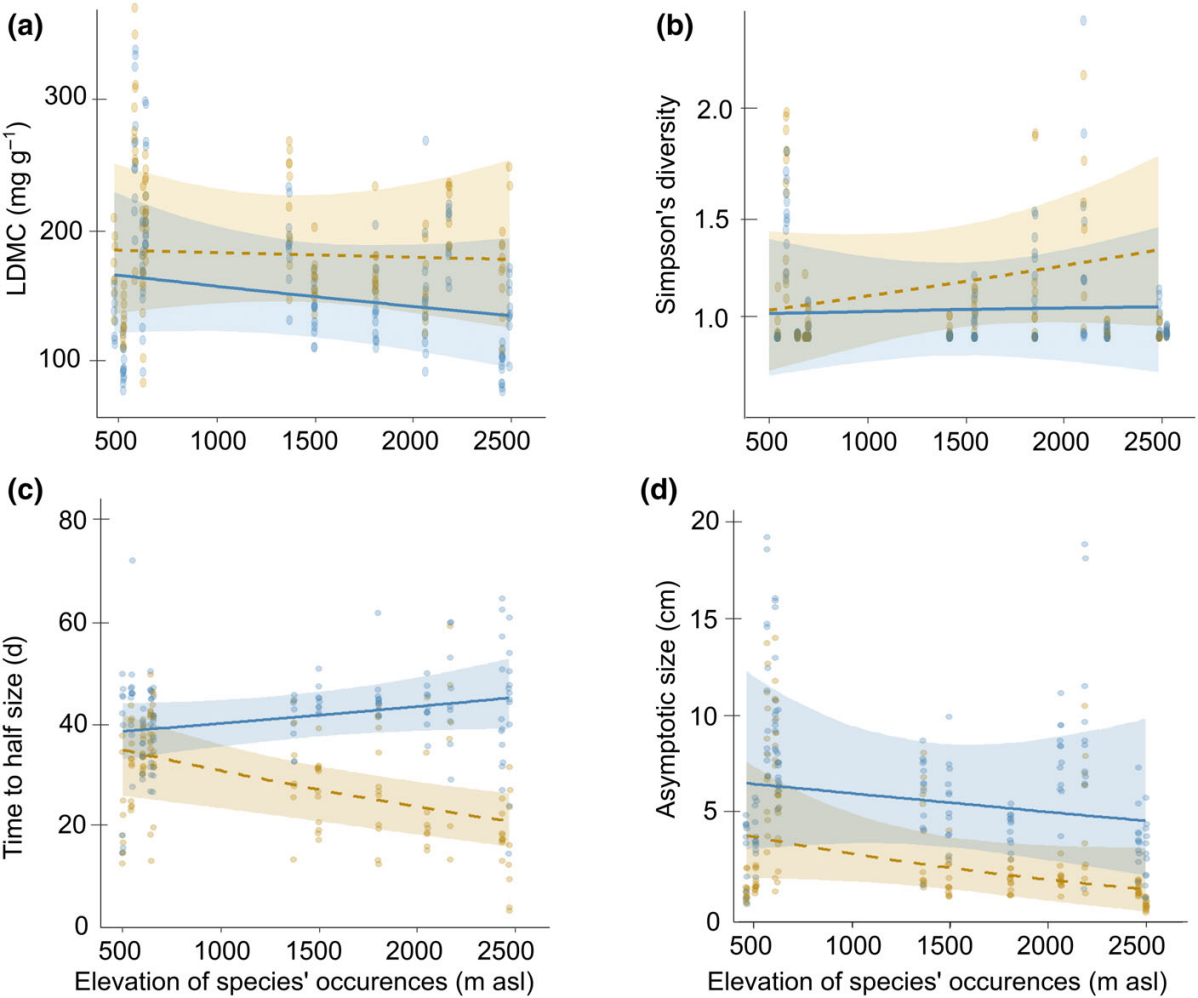
Physical defense, chemical defense, and growth traits in relation to the median elevation of species' occurrences, without or with herbivory‐induction (MeJA treatment) in the climate‐chamber experiment: (a) leaf dry matter content, LDMC; (b) Simpson's diversity index of glucosinolates; (c) time to half size; (d) asymptotic size. Colors represent the herbivory‐induction (MeJA), in blue the control plants (noninduced) and in orange the MeJA‐treated plants (induced), both under benign temperature. The model‐predicted regression lines with 95% confidence limits are indicated (blue and solid lines for control, in brown and dashed lines for MeJA; based on back‐transformed data).

Several interactions between MeJA treatment and elevation of species' occurrences were significant (Table 1; in Fig. 4, comparison to noninduced plants/blue line is of main relevance). Under MeJA treatment as compared to no MeJA, high‐elevation species increased LDMC, lowered SLA, and had higher Simpson's diversity in glucosinolates (Fig. 4a,b). Furthermore, their time to half size (x_mid_) became shorter and their asymptotic size smaller (Fig. 4c,d). Generally speaking, high‐elevation species mounted stronger induced responses than low‐elevation species.

#### Responses to changes in temperature (H5)

**Fig. 5:**
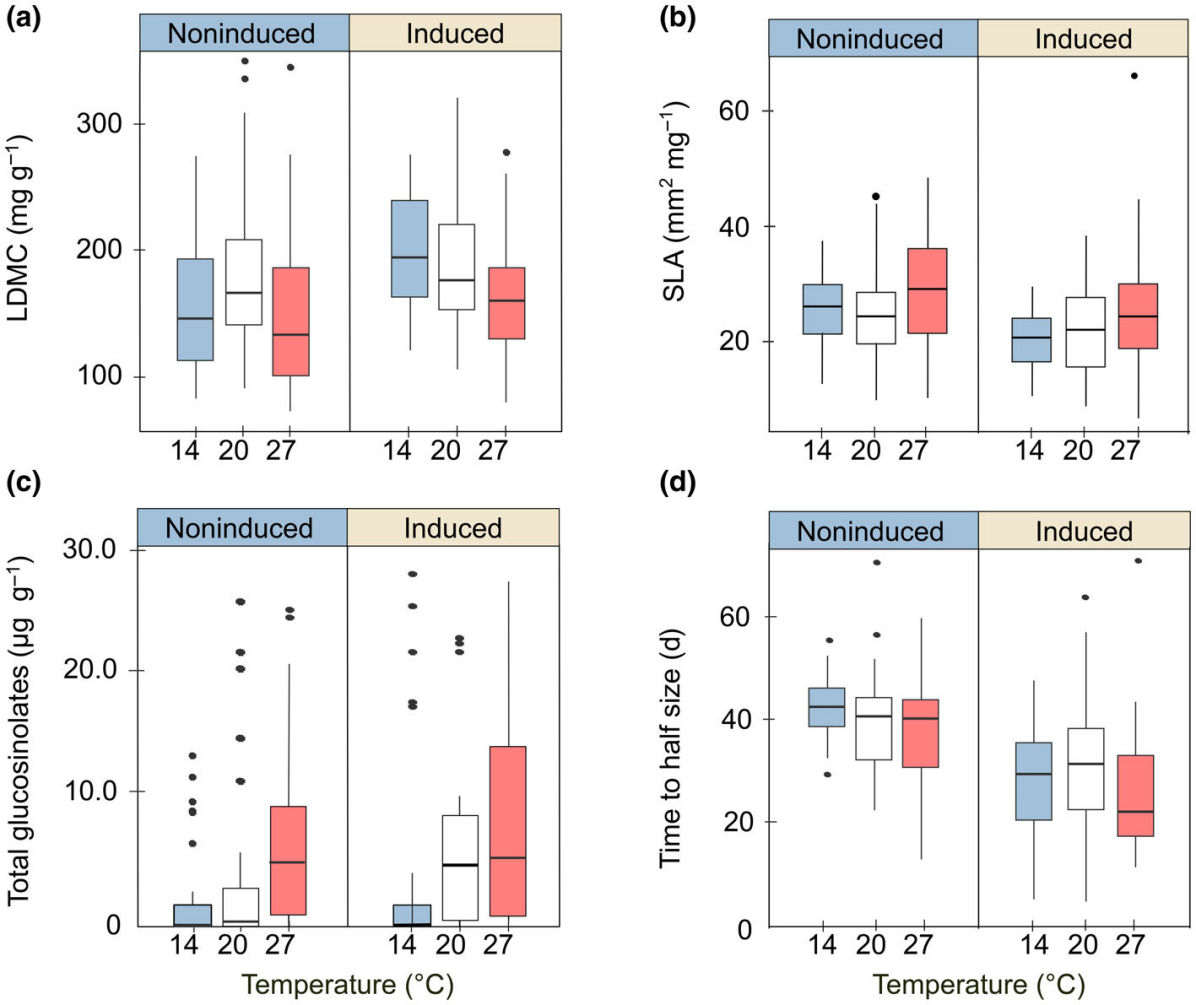
Defense and growth traits in the climate‐chamber experiment, under no herbivory induction (left side of panels) or herbivory induction, and under the three temperature treatments: (a) leaf dry matter content, LDMC; (b) specific leaf area, SLA; (c) total glucosinolate concentration; (d) time to half size. The thick line of boxplots represents the median across plants of the 12 Brassicaceae species, the box indicates the lower and upper quartiles, the whiskers extend to the smallest and largest values within 1.5 times the IQR from the quartiles, and points beyond this range are outliers (untransformed data). The different colors depict the different regimes of temperature used: light blue for cold (14°C), white for benign (20°C), and red for warm (27°C).

Lower and higher than benign temperatures without MeJA induction affected leaf structure and chemical defense traits, suggesting changes in constitutive defense with temperature (Table 1). Cold and warm treatments affected leaf toughness, resulting in lower LDMC and, under warm conditions, higher SLA (Fig. 5a,b, on the left). Furthermore, under cold, concentrations of total and aliphatic glucosinolates – with the latter contributing most to total glucosinolates (*R* ^2^ = 0.96) – were lower, and aromatic and indole glucosinolates remained more often undetected (Fig. 5c, left). In line, the three diversity indices for glucosinolates were significantly lower. In contrast, warm conditions led to increased concentrations of total glucosinolates (Fig. 5c, left), with significant increases in all three chemical families (aliphatic, aromatic, indole), and in glucosinolate richness. Finally, under cold, plants had lower growth rates and were smaller, while under warm conditions, no significant change in growth was detected. Results indicate lower constitutive defense in leaf morphology under colder and warmer relative to benign temperature, and divergent responses of constitutive chemical defense to temperature, a decrease when temperature changes to colder and an increase when it changes to warmer.

Exogenous MeJA application influenced temperature responses. Under colder and warmer conditions compared to benign and the application of MeJA, LDMC increased, and only under cold, SLA decreased, suggesting heightened induced physical defense (Table 1; Fig. 5a,b). Changes in chemical defense were small. Fewer plants had detectable glucosinolate levels under cold (Fig. 5c), and fewer plants had detectable indole glucosinolates under warm conditions. Then, under cold and warm conditions combined with MeJA application, time to half size was earlier (Fig. 5d; for cold only a trend). In summary, temperature responses to induced defense mainly affected leaf structural traits.

## Discussion

Our study addressed how temperature shapes plant defense to herbivores over evolutionary and ecological time scales. As a proxy for a temperature gradient along which plant species had evolved, we used elevation and plant species differing in elevational distribution. First, we investigated whether herbivore abundance decreased along a local elevational gradient and found some evidence in sweep net samples. Second, in an outdoor transplant experiment, we examined differences among low‐ and high‐elevation species in susceptibility to herbivory and detected that high‐elevation plants generally experienced lower levels of herbivory. Third, a climate‐chamber experiment suggested that high‐elevation species were more herbivore‐resistant because they mounted heightened induced defenses under herbivore attack, simulated by MeJA application. Finally, the climate‐chamber experiment indicated strong differences in constitutive and, to a lesser extent, in induced defense under cold and warm as compared to benign temperature. Findings are discussed within the broader framework of evolutionary adaptations to herbivory in plant species from cooler vs warmer climates and temperature‐dependent plastic adjustments.

In a restricted survey over a mountain slope, we examined whether the abundance of likely herbivorous insects declined with elevation. Sweep net sampling yielded fewer potential herbivores at higher than at lower elevations (Fig. 1b), supporting the idea that ectothermic animals become less active and abundant in cooler climates, where lower temperatures slow their metabolism, growth, development, and reproduction (Bale *et al*., 2002). Sweep nets predominantly collected hemipterans, which showed a decline in abundance with elevation, as previously demonstrated (Janzen *et al*., 1976). Coleopterans were less abundant but exhibited a similar pattern in sweep nets. However, these trends of declining herbivore abundance with elevation were not observed in pitfall traps, possibly because the two methods capture different insect groups (Hohbein & Conway, 2018). Indeed, pitfall traps were mostly dominated by coleopterans, which did not seem to decline in abundance with elevation – at least in these traps, as previously observed in the eastern Swiss Alps (Gilgado *et al*., 2022). Although this survey was limited in scope due to the limited number of sampling sites, sampling rounds, and taxonomic resolution, the sweep net results generally align with previous studies showing a decline in herbivore abundance with increasing elevation (Janzen *et al*., 1976; Pellissier *et al*., 2012; Pitteloud *et al*., 2020). However, this pattern did not translate into lower damage levels at higher elevation in our study. We detected no trend of declining herbivory with elevation across the transplant sites.

Given the often lower abundance of herbivores at high elevations, one might expect high‐elevation plant species to be less protected in the face of herbivory, but this was not found. Instead, our field experiment with 30 Brassicaceae species revealed that high‐elevation species incurred less damage from herbivores than low‐elevation species in spring and early summer when exposed to the same environmental conditions (Fig. 2b). These results are consistent with previous studies on Brassicaceae, which showed for *Arabis alpina* L. and 16 *Cardamine* species that those originating from the alpine range were less affected by herbivores than their low‐elevation counterparts (Pellissier *et al*., 2016; Buckley *et al*., 2019). The climate‐chamber experiment provided insight into how high‐elevation species are likely to achieve this better protection. High‐elevation species did not differ from low‐elevation species in leaf‐physical and chemical defense traits in the absence of herbivory induction, indicating no difference in constitutive defense (Table 1). However, under MeJA treatment, which simulated herbivore attack, the high‐elevation species increased their leaf dry matter content more than low‐elevation species, suggesting that they may have acquired some resistance due to sturdier leaves. However, with this increase in leaf dry matter content, high‐elevation species reached about the expression level of low‐elevation species (Fig. 4a), and therefore, this change cannot fully explain their better protection seen in the field.

The trait that was induced and that most likely increased protection of high‐elevation species was glucosinolate expression. Although total glucosinolate amount and compound richness did not significantly differ over the elevational gradient, high‐elevation species exhibited greater evenness in compounds, suggesting a shift in allocation strategy rather than an overall quantitative increase (Fig. 4b). This increase of phytochemical evenness in high‐elevation species may reflect a key component of their induced defense strategy. By a more balanced expression of chemical compounds, plants may enhance the likelihood of producing effective compounds in good concentrations against a broader range of herbivores, as posited by the screening hypothesis (Jones & Firn, 1991; Firn & Jones, 2003). Even though higher elevations may not sustain a broader range of herbivores than lower elevations, this greater potential of chemical defense of alpine species may enhance their overall defense effectiveness. In fact, increasing phytochemical diversity and evenness can be a more efficient and cost‐effective strategy against insect herbivores, as together, compounds may act simultaneously on multiple aspects of herbivore physiology (López‐Goldar *et al*., 2024).

Results are clearly more supportive of some long‐standing hypotheses in the field of plant defense evolution and less supportive of others. For example, we found that high‐elevation species did not exhibit higher constitutive defense (Table 1), providing no support for the resource availability hypothesis. This hypothesis centers around the costs of tissue replacement and posits that under harsh (and nutrient‐poor) conditions, plants should invest in constitutive defense (Coley *et al*., 1985; Endara & Coley, 2011). At high elevation, an especially harsh environmental aspect is the short growing season (Körner, 2003; Patsiou *et al*., 2021), under which tissue replacement must be a challenge. Our findings are more in line with hypotheses favoring induced defense, a strategy predicted to be advantageous against herbivory when it occurs irregularly or when maintaining high levels of constitutive defense comes with considerable costs (Karban *et al*., 1997; Agrawal *et al*., 1999). Herbivore pressure may indeed be rarer in alpine regions (Rasmann *et al*., 2014b).

General allocation hypotheses seem of additional relevance. The smaller size of plants under herbivory induction (Fig. 3d), particularly in high‐elevation species, aligns with the predicted shift in resource allocation from growth to defense (Koricheva *et al*., 2004; Züst *et al*., 2015). Although we found hardly any evidence for correlations between growth and defense traits within treatments (Table S8), this does not rule out the importance of trade‐offs. Also, there is the possibility that the earlier growth of high‐elevation species in response to herbivory induction may partly be an escape strategy that comes with its own trade‐off, smaller size (Fig. 4c,d). Previous macroevolutionary work in Brassicaceae revealed that under heat, speed of growth and size are involved in a trade‐off (Maccagni & Willi, 2022). Namely, under daily heat bouts, high‐elevation species exhibited earlier and faster growth, but their asymptotic size was smaller. Jasmonic acid is a key signaling molecule associated with plant defense, but it also plays a significant role in plant resilience to various abiotic stresses, such as cold, heat, drought, salt, and heavy metals (reviewed in Wang *et al*., 2020a). Therefore, observed patterns of re‐allocation in response to jasmonate treatment could involve escape, possibly even as a by‐product of adaptation to a short growing season, although further investigations are required to confirm this.

Along the elevational gradient, temperature stands out as a key aspect of the abiotic environment that shapes a plant's phenotype and affects plant–herbivore interactions. Our study showed that temperature alone affects constitutive defense. We found that under cold, leaf structural defense (LDMC), and concentrations and diversity of glucosinolates were lower (Table 1; Fig. 5a,c). Under warm conditions, plants still produced weaker and thinner leaves, but glucosinolate concentrations and diversity increased. Additional herbivory induction led to an increase in physical defense both under cold and warm conditions, and it reduced the proportion of plants with detectable chemical defense under cold (Table 1). The effect on phytochemical concentrations and diversity may to some extent reflect the effects of temperature on aspects of the glucosinolate biosynthesis pathway, including gene expression and enzyme activity (Jamieson *et al*., 2012; Wang *et al*., 2020a; Rao *et al*., 2021). However, the increase in chemical defense may also be an evolved response to cope with the enhanced activity of ectothermic herbivores under warm conditions, given the reduced (constitutive) structural defense.

Immediate temperature responses can also be compared with evolved species differences to gain insight into the expression of co‐ or counter‐gradient adaptation (Conover *et al*., 2009). If plastic changes align with evolved differences over a temperature gradient, this suggests adaptive evolution toward reinforcing plastic effects. Conversely, if the patterns of plastic and evolved changes are opposite, it may indicate that maladaptive plasticity was counteracted by adaptive evolution. Since constitutive defense did not differ between low‐ and high‐elevation species, there was no evidence of co‐ or counter‐gradient variation at that level. However, the induced stronger leaf structures and higher evenness in chemical diversity in high‐elevation species and lower expression of leaf structures and chemical diversity under cold reflect counter‐gradient variation.

Overall, our study suggests that induced defense against herbivores is the predominant form of protection in high‐elevation Brassicaceae species. Low‐elevation species are less well protected but may have a higher tolerance and more time to recover after an attack. Our study also highlights the importance of phytochemical diversity and evenness as defense mechanisms in plants, which increase in prevalence at higher elevations. Furthermore, while warmer conditions due to climate change will increase herbivore abundance and activity and consequently increase herbivory pressure on plants (Bale *et al*., 2002; Jamieson *et al*., 2012), we demonstrated that the species studied, including high‐elevation Brassicaceae, can perform well under warmer conditions with possibly more herbivores. Finally, although the jasmonate pathway has primarily been studied for its role in defense against herbivory, our results suggest its central role in mediating the crosstalk between biotic and abiotic stress responses, particularly in high‐elevation plants. We advocate that future research explores the interaction between herbivory and abiotic factors (e.g. temperature) to fully unveil the mechanisms driving the evolution of defense strategies.

## Competing interests

None declared.

## Author contributions

JD, AM, and YW conceived and designed the experiments. JD and AM conducted the experimental work. TD performed the statistical analyses with support by AM and YW. TD and YW wrote the manuscript with contributions from SR, JD, and AM. GG identified the chemical compounds, and SR supported the analysis of raw compound data. All authors gave final approval for publication.

## Disclaimer

The New Phytologist Foundation remains neutral with regard to jurisdictional claims in maps and in any institutional affiliations.

## Supporting information


**Fig. S1** Variation in temperature measured 5 cm aboveground at the transplant sites.
**Fig. S2** Pictures of the experimental setup in the transplant experiment.
**Fig. S3** Best‐supported growth model in the climate‐chamber experiment.
**Fig. S4** Variation in herbivore damage observed in the transplant experiment.
**Methods S1** Species sampling.
**Methods S2** Sowing and pretransplant conditions.
**Methods S3** Herbivore sampling.
**Methods S4** Modeling growth and extracting growth parameters.
**Table S1** List of taxa used in the study.
**Table S2** Data on transplant sites, including dates or periods when assessments were done.
**Table S3** Best‐supported growth model in the climate‐chamber experiment.
**Table S4** Model comparison using LOO cross‐validation.
**Table S5** Transplant experiment: effect of sampling method, elevation of sites or mean annual temperature, and their interaction on herbivore abundance.
**Table S6** Transplant experiment: effect of elevation of sites, median elevation of species' occurrences, and their interaction on herbivore damage.
**Table S7** Climate‐chamber experiment: trait differences among species.
**Table S8** Climate‐chamber experiment: correlation matrix on traits measured under benign temperature in the control and herbivory‐induced treatments.
Please note: Wiley is not responsible for the content or functionality of any Supporting Information supplied by the authors. Any queries (other than missing material) should be directed to the *New Phytologist* Central Office.

## Data Availability

Data of the transplant experiment and the climate‐chamber experiment are saved on Zenodo (doi: 10.5281/zenodo.17249195).
