# Supplementary material for: Variation in herbivore defense strategies among plant species differing in elevational distribution and the role of temperature in defense
Source: New Phytol. 2026 Jan 7;249(6):3091–103. doi: 10.1111/nph.70872 (PMC12917448; doi:10.1111/nph.70872)
Supplement: Supplementary file 1 — Fig. S1 Variation in temperature measured 5 cm aboveground at the transplant sites. Fig. S2 Pictures of the experimental setup in the transplant experiment. Fig. S3 Best‐supported growth model in the climate‐chamber experiment. Fig. S4 Variation in herbivore damage observed in the transplant experiment. Methods S1 Species sampling. Methods S2 Sowing and pretransplant conditions. Methods S3 Herbivore sampling. Methods S4 Modeling growth and extracting growth parameters. Table S1 List of taxa used in the study. Table S2 Data on transplant sites, including dates or periods when assessments were done. Table S3 Best‐supported growth model in the climate‐chamber experiment. Table S4 Model comparison using LOO cross‐validation. Table S5 Transplant experiment: effect of sampling method, elevation of sites or mean annual temperature, and their interaction on herbivore abundance. Table S6 Transplant experiment: effect of elevation of sites, median elevation of species' occurrences, and their interaction on herbivore damage. Table S7 Climate‐chamber experiment: trait differences among species. Table S8 Climate‐chamber experiment: correlation matrix on traits measured under benign temperature in the control and herbivory‐induced treatments. Please note: Wiley is not responsible for the content or functionality of any Supporting Information supplied by the authors. Any queries (other than missing material) should be directed to the New Phytologist Central Office. [file NPH-249-3091-s001.pdf]

***New Phytologist* Supporting Information**

Article title: Variation in herbivore defense strategies among plant species differing in elevational distribution, and the role of temperature in defense
Authors: Thomas Dorey, Janisse Deluigi, Alessio Maccagni, Sergio Rasmann, Gaétan Glauser, Yvonne Willi
Article acceptance date: 11 December 2025

The following Supporting Information is available for this article:

Fig. S1 Variation in temperature measured 5 cm above ground at the transplant sites.

Fig. S2 Pictures of the experimental setup in the transplant experiment.

Fig. S3 Best-supported growth model in the climate-chamber experiment.

Fig. S4 Variation in herbivore damage observed in the transplant experiment.

Table S1 List of taxa used in the study.

Table S2 Data on transplant sites, including dates or periods when assessments were done.

Table S3 Best-supported growth model in the climate-chamber experiment.

Table S4 Model comparison using LOO cross-validation.

Table S5 Transplant experiment: effect of sampling method, elevation of sites or mean annual temperature, and their interaction on herbivore abundance.

Table S6 Transplant experiment: effect of elevation of sites, median elevation of species' occurrences, and their interaction on herbivore damage.

Table S7 Climate-chamber experiment: trait differences among species.

Table S8 Climate-chamber experiment: correlation matrix on traits measured under benign temperature in the control and herbivory-induced treatments.

Methods S1 Species sampling.

Methods S2 Sowing and pre-transplant conditions.

Methods S3 Herbivore sampling.

Methods S4 Modeling growth and extracting growth parameters.

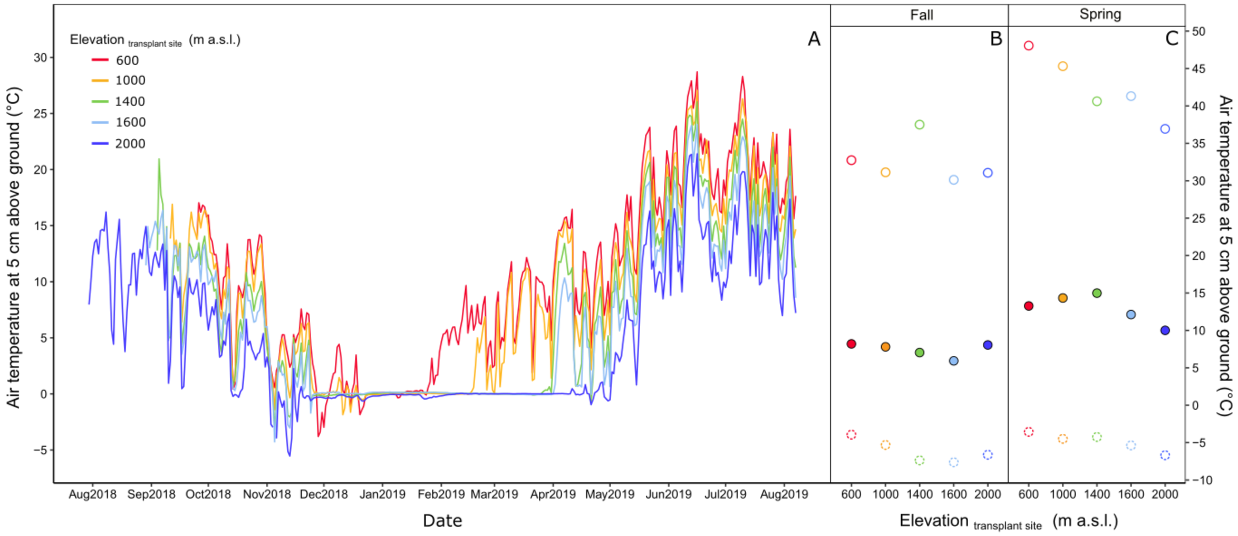

Figure S1: Variation in temperature measured 5 cm above ground at the transplant sites: A. daily mean temperature for the whole duration of the experiment; B. averaged daily mean temperature (middle) as well as absolute minimum (dashed open circles on bottom) and absolute maximum (open circles on top) over the period of assessing leaf damage in fall 2018, and C. in spring 2019. Fall and spring were defined by temperature change (see Table S2 for dates). The different colors indicate the five transplant sites.

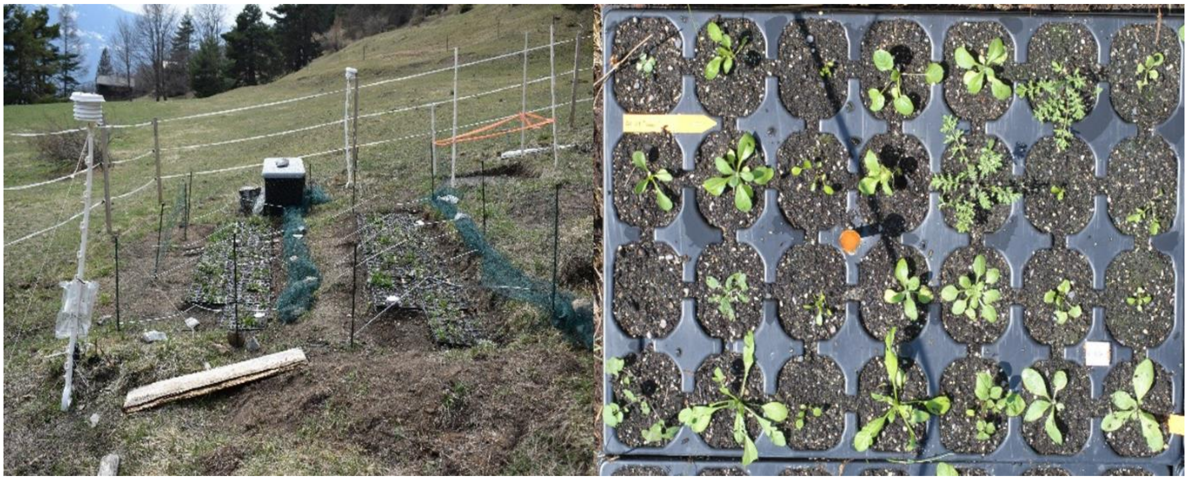

Figure S2: Pictures of the experimental setup in the transplant experiment. At each site, we planted roughly 600 plants of 30 Brassicaceae species varying in elevational distribution, each from (max.) 2 populations, with 5 families per population, and 2 replicates per family arranged in two spatial blocks (left). Within blocks, plants grew in multipot trays buried into the soil up to the rims (right). A 1m-high fence was installed to protect the plants from large herbivores.

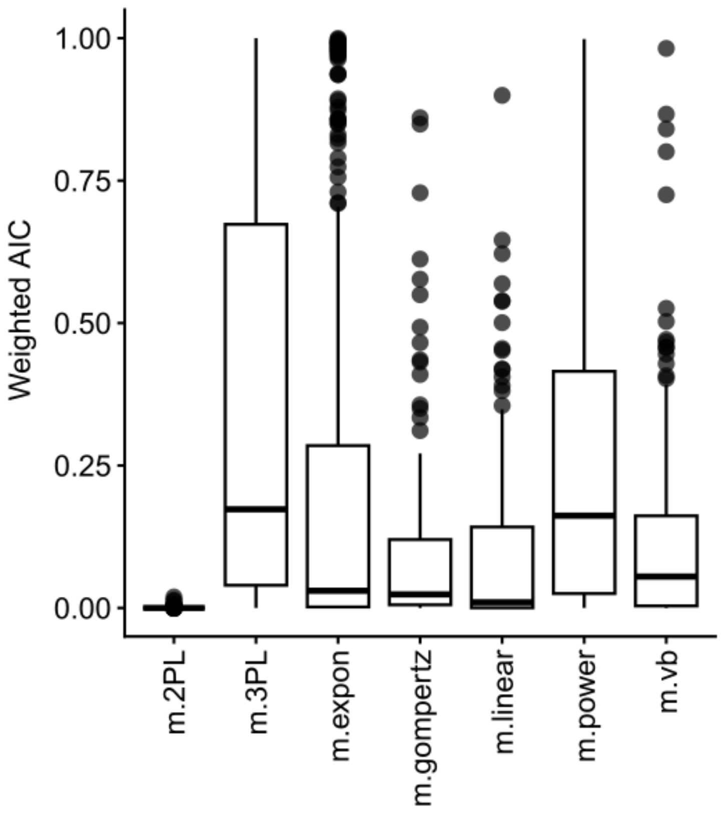

Figure S3: Best-supported growth model in the climate-chamber experiment. Boxplots show the distribution of weighted AIC values for each of the seven growth models across all plants (the thick line represents the median, the box shows the upper and lower quartiles, the whiskers extend to the smallest and largest values within 1.5 times the IQR from the quartiles, and points beyond this range are outliers). The average length of the two longest leaves of each plant was measured regularly and used for fitting individual growth models: 2- and 3-parameter logistic, exponential, Gompertz, linear, power, and von Bertalanffy. The 3-parameter logistic model got the highest support. Growth parameters (x_{mid}/time when half the asymptotic size was reached, maximal growth rate, and asymptotic size) were extracted and used for statistical analyses.

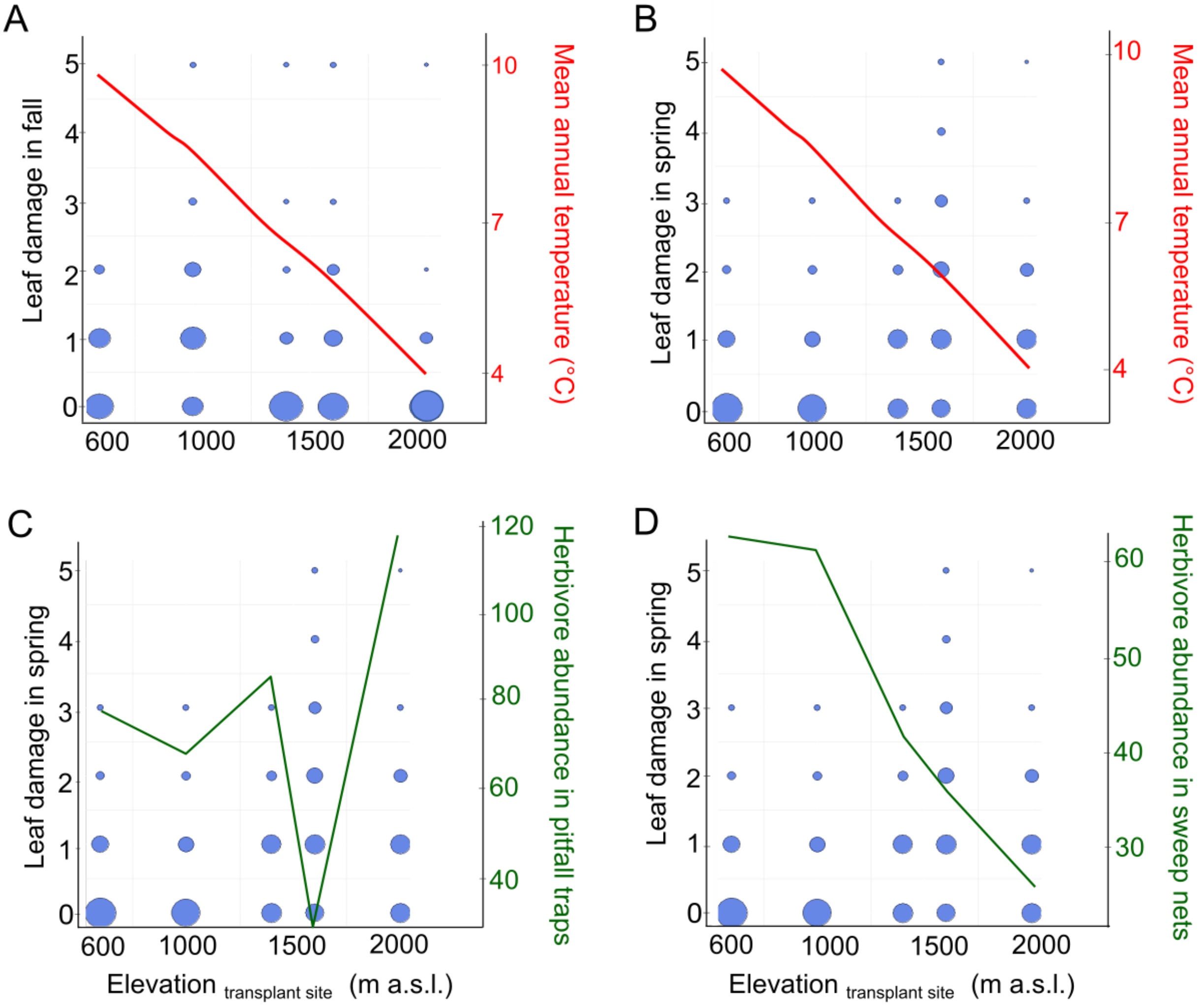

Figure S4: Variation in herbivore damage observed in the transplant experiment: A. in fall, and B-D. in spring over the five transplant sites of the elevational gradient. The size of circles represents the number of plants with a particular damage level at a specific elevation. Additionally, the panels contain information on mean annual temperature of transplant sites (in red in panels A and B), and mean herbivore abundance measured weekly (in green) in pitfall traps (C) and by sweep net sampling (D).

Table S1: List of taxa (species and subspecies) used in the study. The plant species names are based on the Infoflora database (www.infoflora.ch, v.2020). Seeds were collected in Switzerland from two different sites at least 50km apart from each other (i.e., population) at an elevation typical for the species. Seeds were harvested from separate mother plants in the field (i.e., family). The median elevation of species is based on occurrence data from Patsiou *et al.* (2021). All 30 plant species were used in the field experiment. Life-form information is based on the Raunkiaer classification (Raunkiaer 1934). The subset of 12 plant species used for the climate-chamber experiment are indicated in bold.

Plant species	Location (Canton)	Coordinates (° N, ° E)	Sampling date	Population ID	Seed family ID	Median elevation (m a.s.l.)	Life form
Arabidopsis thaliana L.	Buseno (GR)	47.372; 7.709	21.06.2015	28	4, 5, 6, 7, 8	535	Therophyte
	Basel (BS)	47.583; 7.589	10.05.2017	209	2, 6, 7, 8, 9		
Arabis alpina* subsp. *alpina L.	Medels (GR)	46.582; 8.804	10.08.2016	103	3, 4, 5, 8, 10	1810	Chamaephyte
	Wildhaus Alt St. Johann (SG)	47.242; 9.359	15.09.2016	151	2, 5, 6, 9, 15		
Arabis bellidifolia subsp. *stellulata* Bertol.	Val Müstair (GR)	46.552; 10.421	29.08.2017	228	6, 7, 8, 11, 15	2263	Perennial hemicryptophyte
	Obergoms (VS)	46.472; 8.382	28.09.2017	237	1, 2, 6, 14, 18		
Arabis caerulea All.	Zermatt (VS)	46.024; 7.805	13.09.2016	147	4, 6, 8, 10, 15	2640	Perennial hemicryptophyte
	Lischana / Scuol (GR)	46.761; 10.343	19.09.2016	162	1, 6, 10, 11, 13		
Arabis ciliata Clairv.	Zermatt (VS)	46.013; 7.795	27.08.2016	144	2, 6, 8, 13, 15	1472	Therophyte
	Capriasca (TI)	46.103; 8.964	12.06.2017	211	3, 5, 6, 7, 20		
Arabis collina Ten.	Pazzallo (TI)	45.977; 8.947	10.07.2017	215	2, 4, 5, 6, 8, 9, 10, 12, 13, 14	516	Therophyte
Arabis nova Vill.	Grächen (VS)	46.194; 7.826	15.07.2016	94	2, 5, 6, 8, 12	1036	Therophyte
	Poschiavo (GR)	46.315; 10.071	20.07.2016	111	4, 5, 8, 13, 14		
Arabis sagittata Bertol.	Rovio (TI)	45.941; 8.998	09.07.2015	16	2, 6, 7, 11, 15	400	Therophyte
	Montcherand (VD)	46.729; 6.494	28.08.2016	138	3, 4, 5, 6, 7		
Barbarea vulgaris R. Br.	Mendrisio (TI)	45.913; 9.004	09.08.2016	125	5, 6, 10, 11, 13	541	Therophyte
	Hauterive (FR)	46.770; 7.121	23.07.2016	102	2, 4, 9, 12, 13		
Capsella bursa-pastoris L.	Pambio-Noranco (TI)	45.981; 8.928	05.05.2016	26	6, 8, 9, 11, 12	559	Therophyte
	Pfungen (ZH)	47.518; 8.628	09.06.2016	55	2, 4, 6, 11, 15		
Capsella rubella Reut.	Melano (TI)	45.924; 8.980	27.05.2016	27	3, 4, 7, 8, 10	466	Therophyte
	Fribourg (FR)	46.815; 7.157	15.05.2016	30	3, 5, 7, 9, 13		
Cardamine alpina Willd.	Poschiavo (GR)	46.395; 10.035	02.09.2016	119	4, 7, 8, 10, 12	2490	Perennial hemicryptophyte
	Bedretto (TI)	46.479; 8.390	28.09.2017	239	4, 7, 8, 11, 14		

Cardaminopsis arenosa L.	Fribourg (FR)	46.799; 7.163	16.06.2015	6	14, 18, 33, 37, 41	706	Perennial hemicryptophyte
	Airolo (TI)	46.528; 8.626	29.06.2015	13	8, 10, 11, 18, 27		
Cardamine hirsuta L.	Neuchâtel (NE)	47.000; 6.934	28.04.2015	1	9, 12, 13, 35, 49	542	Therophyte
	Zug (ZG)	47.178; 8.493	05.05.2015	2	3, 4, 15, 37, 48		
Cardamine impatiens L.	Neunkirch (SH)	47.671; 8.494	03.07.2016	57	3, 5, 6, 7, 11	565	Therophyte
	Castel San Pietro (TI)	45.860; 9.016	03.06.2017	207	6, 18, 24, 28, 30		
Cardamine resedifolia L.	Zermatt (VS)	46.014; 7.822	06.08.2016	95	3, 6, 7, 13, 15	2360	Perennial hemicryptophyte
	Poschiavo (GR)	46.408; 10.007	05.08.2016	113	4, 9, 14, 16, 17		
Cardaminopsis halleri L.	Beride (TI)	46.003; 8.834	12.06.2016	115	7, 12, 13, 15, 16	1141	Subfructicose chamaephyte
	St. Moritz (GR)	46.501; 9.852	10.08.2016	44	2, 4, 6, 7, 11		
Descurainia sophia L.	Äscher Wildkirchli (Al)	47.283; 9.413	11.07.2016	77	4, 5, 7, 12, 14	1028	Therophyte
	Sion (VS)	46.220; 7.349	06.06.2016	24	3, 8, 10, 12, 15		
Draba dubia Suter	Quinto (TI)	46.533; 8.677	19.07.2016	75	1, 2, 3, 4, 5	2440	Subfructicose chamaephyte
	Zermatt (VS)	45.983; 7.781	14.09.2016	150	5, 10, 11, 12, 16		
Draba muralis L.	Maroggia (TI)	45.934; 8.972	15.04.2017	196	3, 5, 8, 9, 17	497	Therophyte
	Uster (ZH)	47.340; 8.752	21.06.2017	202	1, 3, 4, 5, 9		
Draba siliquosa M. Bieb.	Zermatt (VS)	46.015; 7.791	07.08.2016	81	5, 6, 7, 11, 12	2568	Perennial hemicryptophyte
	Pontresina (GR)	46.414; 10.008	04.08.2016	122	6, 7, 9, 11, 13		
Erophila praecox Steven	Fribourg (FR)	46.819; 7.157	17.05.2016	31	2, 6, 8, 12, 14	445	Therophyte
Fourraea alpina L.	Arzo (TI)	45.883; 8.948	09.07.2016	221	2, 3, 8, 9, 12	1421	Therophyte
	San Bernardino (GR)	46.464; 9.206	15.08.2017	68	2, 3, 11, 13, 14		
Hugueninia tanacetifolia L.	Bourg St. Pierre (VS)	45.870; 7.169	04.10.2016	140	4, 5, 6, 7, 8, 9, 11, 12, 13, 14	1997	Geophyte
Kernera saxatilis L.	Seewis (GR)	46.977; 9.498	04.07.2016	65	2, 3, 9, 11, 14	1550	Perennial hemicryptophyte
	Pazzallo (TI)	45.977; 8.947	19.06.2017	213	3, 4, 11, 16, 21		
Pritzelago alpina subsp. *brevicaulis* Spreng.	Zermatt (VS)	45.983; 7.781	28.08.2016	146	2, 9, 11, 13, 14	2791	Perennial hemicryptophyte
	Lischana / Scuol (GR)	46.761; 10.343	19.09.2016	164	2, 3, 4, 7, 9		
Thlaspi brachypetalum Jord.	Amsteg (UR)	46.748; 8.644	09.06.2015	5	12, 14, 41, 46, 50	1104	Monocarpic hemicryptophyte
	Escholzmatt (LU)	46.921; 7.923	20.06.2016	48	8, 12, 13, 14, 15		
Thlaspi perfoliatum L.	Ayent (VS)	46.273; 7.406	28.04.2016	205	4, 5, 15, 17, 18	585	Therophyte
	Meride (TI)	45.901; 8.94	30.04.2017	21	2, 3, 6, 7, 9		
Thlaspi rotundifolium subsp. *corymbosum* Gremlin	San Vittore (GR)	46.299; 9.068	14.08.2016	106	2, 9, 10, 11, 13	2264	Subfructicose chamaephyte
	Zermatt (VS)	45.985; 7.685	08.09.2016	243	1, 4, 5, 7, 9		
Turritis glabra L.	Mont Noble (VS)	46.194; 7.451	19.08.2016	98	3, 6, 9, 10, 12	785	Monocarpic hemicryptophyte
	Tengia (TI)	46.472; 8.830	22.08.2017	223	2, 4, 5, 6, 9		

Table S2: Data on transplant sites, including dates or periods when assessments were done. Mean annual temperature (MAT) of transplant sites was estimated 5 cm above ground, for the period from October 09, 2018, to August 21, 2019, when temperature was recorded at all sites.

Site, m a.s.l.	Coordinates (° N; ° E)	MAT, °C ± SD	Transplant date	Fall period	Winter period	Spring period	Leaf damage assessment, fall	Leaf damage assessment, spring	Insect sampling, summer
601	46.872; 9.519	10.20 ± 7.97	11.10.2018	11.10.2018 to 10.12.2018	11.12.2018 to 23.01.2019	24.01.2019	14.10.2018 to 02.12.2018	05.03.2019 to 23.04.2019	14.05.2019 to 30.07.2019
997	46.874; 9.509	8.66 ± 7.86	27.09.2018	27.09.2018 to 14.12.2018	15.12.2018 to 19.03.2019	20.03.2019	05.10.2018 to 23.11.2018	01.05.2019 to 19.06.2019	21.05.2019 to 13.08.2019
1395	46.869; 9.490	6.73 ± 7.60	20.09.2018	20.09.2018 to 07.12.2018	08.12.2018 to 05.05.2019	06.05.2019	27.09.2018 to 15.11.2018	16.06.2019 to 04.08.2019	29.05.2019 to 13.08.2019
1610	46.878; 9.495	6.00 ± 7.01	14.09.2018	14.09.2018 to 24.12.2018	25.12.2018 to 13.04.2019	14.04.2019	19.09.2018 to 07.11.2018	20.05.2019 to 08.07.2019	04.06.2019 to 13.08.2019
1998	46.888; 9.489	4.08 ± 6.20	15.08.2018	15.08.2018 to 13.11.2018	14.11.2018 to 27.04.2019	28.04.2019	20.08.2018 to 08.10.2018	03.06.2019 to 22.07.2019	26.06.2019 to 27.08.2019

Table S3: Best-supported growth model out of seven (linear, exponential, power, 2- and 3-parameter logistic, Gompertz, and von Bertalanffy) for each species (abbreviated by the first letter of the genus name and the first three letters of the species name) under the six treatment combinations (temperature and herbivory induction) in the climate-chamber experiment. Weighted AIC (AIC_w)is the relative performance of a particular model for a plant; the median across the replicate plants is reported. K represents the number of plants of a species in a treatment combination. The 3-parameter logistic model had the highest support across all species and treatment combinations.

	Cold, no herbivory induction			*Cold, herbivory induction*			*Benign, no herbivory induction*			*Benign, herbivory induction*			*Warm, no herbivory induction*			*Warm, herbivory induction*		
Species	Model	K	AIC_w median	Model	K	AIC_w median	Model	K	AIC_w median	Model	K	AIC_w median	Model	K	AIC_w median	Model	K	AIC_w median
Aalp	m.expon	3	0.527	m.power	3	0.670	m.3PL	4	0.837	m.power	3	0.474	m.power	3	0.489	m.3PL	4	0.407
Acil	m.expon	3	0.731	m.power	3	0.459	m.power	3	0.693	m.3PL	4	0.467	m.power	3	0.512	m.vb	4	0.303
Acol	m.expon	3	0.823	m.3PL	4	0.761	m.power	3	0.664	m.3PL	4	0.334	m.expon	3	0.379	m.gompertz	4	0.441
Bvul	m.3PL	4	0.840	m.power	3	0.309	m.3PL	4	0.972	m.3PL	4	0.832	m.3PL	4	0.913	m.3PL	4	0.658
Calp	m.expon	3	0.401	m.power	3	0.482	m.expon	3	0.726	m.power	3	0.555	m.linear	3	0.378	m.vb	4	0.378
Cbur	m.3PL	4	0.711	m.3PL	4	0.476	m.3PL	4	0.962	m.3PL	4	0.939	m.3PL	4	0.839	m.3PL	4	0.554
Chal	m.expon	3	0.888	m.vb	4	0.307	m.power	3	0.546	m.3PL	4	0.391	m.power	3	0.464	m.power	3	0.231
Cimp	m.power	3	0.368	m.3PL	4	0.348	m.3PL	4	0.783	m.3PL	4	0.680	m.3PL	4	0.705	m.power	3	0.278
Dmur	m.expon	3	0.884	m.power	3	0.429	m.3PL	4	0.814	m.linear	3	0.292	m.expon	3	0.297	m.linear	3	0.359
Dsil	m.3PL	3	0.737	m.gompertz	4	0.444	m.expon	3	0.457	m.power	3	0.316	m.power	3	0.319	m.3PL	4	0.308
Dsop	m.expon	3	0.378	m.power	3	0.912	m.gompertz	4	0.435	m.power	3	0.610	m.3PL	4	0.353	m.power	4	0.392
Htan	m.3PL	4	0.271	m.power	3	0.380	m.power	3	0.338	m.expon	3	0.879	m.expon	3	0.686	NA	NA	NA

Table S4: Model comparison using leave-one-out (LOO) cross-validation (elpd_loo ± SE) for each trait (N represents sample size). The "full model" includes all main effects and interactions: trait ~ elevation of species' occurrences × herbivory induction × temperature. A first reduced model omitted the three-way interaction; a second both the three-way interaction and the elevation of species' occurrences × temperature interaction. Following Vehtari et al. (2017), we considered a difference in LOOIC to indicate a meaningful improvement in predictive accuracy if the estimated difference exceeded its standard error. In all cases, differences between models were smaller than this threshold, indicating similar performance. Accordingly, the most parsimonious model was retained (indicated in bold).

Traits	N	Full model	No three-way interaction	No three-way interaction and no elevation of species x temperature interaction
**Defense traits**				
Leaf dry matter content	248	268.9 ± 14.4	271.4 ± 14.2	**271.5 ± 14.2**
Specific leaf area	247	188.4 ± 15.9	191.7 ± 16.0	**191.5 ± 15.9**
Total conc. glucosinolates	250	-1872.7 ± 59.7	-1858.4 ± 61.6	**-1859.3 ± 61.6**
Conc. aliphatic glucosinolates	250	-1786.7 ± 60	-1783.9 ± 61.5	**-1783.7 ± 61.5**
Conc. aromatic glucosinolates	250	-883.4 ± 56.4	-877.4 ± 56.3	**-878.3 ± 56.4**
Conc. indole glucosinolates	250	-731.3 ± 43.6	-719.3 ± 43.1	**-718.9 ± 43.1**
Glucosinolate richness	250	133.2 ± 14.1	134.3 ± 14.3	**133.5 ± 14.1**
Simpson's diversity index	250	391.8 ± 45.1	391.9 ± 45.6	**387.8 ± 47.7**
Rao's quadratic entropy	250	35.0 ± 22.4	40.3 ± 21.4	**38.8 ± 21.6**
**Growth traits**				
Time to half size	244	119.9 ± 17.4	123.4 ± 17.6	**123.7 ± 17.7**
Maximal growth rate	244	728.2 ± 21.7	724.9 ± 21.8	**724.8 ± 21.8**
Asymptotic size	284	143.2 ± 15.9	145.2 ± 15.9	**145.0 ± 15.9**

Table S5: Transplant experiment: effect of sampling method, elevation of sites or mean annual temperature (MAT, measured during the experiment), and their interaction on herbivore abundance. Sample sizes (N), conditional R^2 and coefficients of fixed effects, the median and 90% high density interval [HDI]) of the posterior distribution are reported. Significant independent variables are indicated in bold (HDI not overlapping with 0, and probability of direction (pd) > 97.5% [(.) >95%, * >97.5%, ** >99.5%, *** >99.95%]). Results for random effects are not shown.

Independent variables		Herbivore abundance
	R_c^2 = 0.47	N = 30
Sampling method		**-0.34 [-0.34, -0.07]*** **
Elevation of transplant sites (ESites)		0.01 [0.00, 0.00]
Sampling method × ESites		**-0.00 [-0.01, 0.00]***
	R_c^2 = 0.45	N = 30
Sampling method		**-0.21 [-0.34, -0.07]*** **
MAT at transplant sites (MATSites)		-0.00 [-0.14, 0.11]
Sampling method × MATSites		0.14 [-0.02, 0.14]

Table S6: Transplant experiment: effect of elevation of transplant sites, median elevation of species' occurrences, and their interaction on herbivore damage in fall 2018 and spring 2019. In another model applied to spring data, we tested whether the abundance of herbivores by sweep net sampling and in pitfall traps instead of elevation could explain damage. The third set of models replaced elevation of transplant sites with mean annual temperature (MAT) at the sites, as measured over the course of the experiment. Sample sizes (N), conditional R^2 and coefficients of fixed effects, the median and 90% high density interval [HDI]) of the posterior distribution are reported. Significant independent variables are indicated in bold (HDI not overlapping with 0, and probability of direction (pd) > 97.5% [(.) >95%, * >97.5%, ** >99.5%, *** >99.95%]). Results for random effects are not presented.

Independent variables	Leaf herbivore damage	
	Fall	Spring
	R_c^2 = 0.39, N = 2763	R_c^2 = 0.38, N = 2053
Elevation of transplant sites (ESites)	-0.37 [-1.94, 1.20]	0.64 [-1.02, 2.29]
Elevation of species' occurrences (ESpecies)	-0.69 [-1.27, -0.09](.)	**-1.00 [-1.80, -0.27]***
ESites × ESpecies	-0.62 [-0.14, 1.40]	0.39 [-0.42, 1.22]
		R_c^2 = 0.39, N = 2053
Herbivore abundance in sweep nets (SWPN)		-0.02 [-0.03, -0.02]
Herbivore abundance in pitfall traps (PFT)		-0.00 [-0.01,0.00]
Elevation of species' occurrences (ESpecies)		**-1.02 [-1.74, -0.25]***
SWPN × ESpecies		**-0.01 [-0.01, -0.00]***
PFT × ESpecies		-0.06 [-0.81,0.68]
	R_c^2 = 0.39, N = 2763	R_c^2 = 0.38, N = 2053
MAT at transplant sites (MATSites)	0.23 [-0.18, 0.61]	-0.25 [-0.56, 0.12]
Elevation of species' occurrences (ESpecies)	-0.68 [-1.27, -0.07](.)	**-1.01 [1.71, 0.20]***
MATSites × ESpecies	-0.09 [-0.16, 0.00]	-0.09 [-0.17, -0.01]

Table S7: Climate-chamber experiment: trait differences (mean ± standard deviation of non-transformed estimates; N indicates the sample size) among plants of the different herbivory-induction and temperature treatments.

	Control/no herbivory induction						Herbivory induction					
	Cold		Benign		Warm		Cold		Benign		Warm	
**Defense traits**	**N**	Mean ± SD	**N**	Mean ± SD	**N**	Mean ± SD	**N**	Mean ± SD	**N**	Mean ± SD	**N**	Mean ± SD
Leaf dry matter content (mg g^{-1})	48	158.41 ± 51.08	44	180.53 ± 67.61	43	148.19 ± 62.08	44	198.32 ± 41.42	40	190.85 ± 56.01	29	176.67 ± 72.30
Specific leaf area (mm^2 mg^{-1})	48	22.50 ± 6.35	44	22.15 ± 9.26	43	27.10 ± 9.66	43	16.61 ± 5.31	40	19.64 ± 8.01	29	23.02 ± 13.09
Conc. total glucosinolates (µg g^{-1})	48	1.92 ± 3.71	44	3.13 ± 6.26	43	6.37 ± 6.69	46	3.38 ± 7.83	40	5.29 ± 6.66	29	7.81 ± 8.08
Conc. aliphatic glucosinolates (µg g^{-1})	48	1.65 ± 3.24	44	2.87 ± 5.88	43	5.54 ± 6.19	46	3.07 ± 7.37	40	4.04 ± 5.51	29	6.90 ± 7.51
Conc. aromatic glucosinolates (µg g^{-1})	48	0.18 ± 0.76	44	0.09 ± 0.28	43	0.56 ± 1.13	46	0.14 ± 0.52	40	1.02 ± 4.00	29	0.57 ± 1.57
Conc. indole glucosinolates (µg g^{-1})	48	0.02 ± 0.06	44	0.02 ± 0.03	43	0.05 ± 0.06	46	0.02 ± 0.07	40	0.06 ± 0.19	29	0.04 ± 0.05
Glucosinolate richness	48	3.94 ± 3.08	44	21.36 ± 22.81	43	29.60 ± 20.17	46	10.37 ± 16.16	40	21.63 ± 20.05	29	21.97 ± 19.53
Simpson's diversity index	48	1.05 ± 0.12	44	1.18 ± 0.40	43	1.18 ± 0.34	46	1.19 ± 0.60	40	1.19 ± 0.54	29	1.36 ± 0.76
Rao's quadratic entropy	48	0.12 ± 0.16	44	0.18 ± 0.24	43	0.27 ± 0.23	46	0.15 ± 0.20	40	0.23 ± 0.24	29	0.26 ± 0.26
**Growth traits**												
Time to half size (day)	39	41.11 ± 6.09	42	40.19 ± 9.88	44	37.81 ± 11.50	40	27.65 ± 10.80	43	29.46 ± 12.00	37	23.56 ± 9.84
Maximal growth rate	39	0.09 ± 0.02	42	0.11 ± 0.05	44	0.11 ± 0.05	40	0.07 ± 0.03	43	0.10 ± 0.07	37	0.14 ± 0.15
Asymptotic size (cm)	48	5.36 ± 2.36	47	6.83 ± 3.26	48	7.53 ± 5.09	47	2.86 ± 2.58	48	4.16 ± 3.52	46	3.41 ± 3.53

Table S8: Climate-chamber experiment: correlation matrix on traits measured under benign temperature in the control and herbivory-induced treatments. All variables are $\log_{10}(x+1)$ transformed. Person correlations on control plants (N=48) are indicated in blue, whereas correlations on MeJA-treated plants (N=47) are shown in orange. Significant correlations are written in bold (with Bonferroni correction, $\alpha = 0.05/66$).

	LDMC	SLA	Total conc. glucosinolates	Conc. aliphatic glucosinolates	Conc. aromatic glucosinolates	Conc. aliphatic glucosinolates	Glucosinolate richness	Simpson's diversity index	Rao's quadratic entropy	Time to half size	Maximal growth rate	Asymptotic size
LDMC		-0.28	-0.48	-0.36	-0.29	0.02	-0.03	-0.40	0.33	0.07	-0.05	0.49
SLA	**-0.53**		0.20	0.06	0.26	0.33	-0.43	-0.19	-0.23	0.13	-0.02	0.06
Total conc. glucosinolates	-0.32	-0.30		-0.47	-0.17	-0.11	-0.26	-0.18	0.34	0.17	-0.12	-0.34
Conc. aliphatic glucosinolates	-0.35	-0.26	-0.38		-0.19	0.24	0.44	0.42	-0.08	0.32	0.00	-0.19
Conc. aromatic glucosinolates	-0.08	-0.30	-0.18	0.38		-0.10	-0.05	0.01	-0.15	-0.27	-0.23	-0.33
Conc. indole glucosinolates	0.36	**-0.62**	-0.34	0.32	0.21		-0.02	-0.04	0.09	0.21	0.03	0.05
Glucosinolate richness	0.00	**-0.50**	-0.54	**0.73**	0.43	**0.75**		0.39	**0.62**	-0.08	-0.01	-0.39
Simpson's diversity index	-0.17	-0.34	-0.24	**0.68**	0.22	0.23	**0.54**		0.02	-0.18	**0.69**	-0.23
Rao's quadratic entropy	0.24	-0.44	-0.44	0.17	0.25	**0.81**	**0.73**	0.20		0.32	0.02	0.13
Time to half size	**-0.78**	0.39	0.37	0.38	0.14	-0.16	0.19	0.19	-0.03		0.06	**0.65**
Maximal growth rate	**0.60**	-0.46	-0.23	0.25	-0.16	0.44	0.05	-0.16	0.29	**-0.57**		0.25
Asymptotic size	**0.58**	-0.36	-0.20	-0.21	-0.19	0.23	-0.05	0.04	0.13	-0.13	**0.61**	

Methods S1 Species sampling. For each of the plant species, seeds were collected at two different sites (i.e., populations, see Table S1) in Switzerland, between March and September of three years (2015-2017). Sites were located in two different main watersheds, likely reflecting separate glacial histories, and they were at least 50 km apart. Furthermore, each sampling site was at an elevation typical for the species. Seeds from replicate mother plants in the field (referred to as "family") were collected and stored separately in paper bags (80 g m-2, 60 × 90 × 12 mm; ELCO AG, Brugg, Switzerland) under cold (4 °C), dark and dry (adding of silica gel) conditions until sowing.

Methods S2 Sowing and pre-transplant conditions. Seeds were sown about two months prior to transplanting in the field and kept in climate chambers, in summer/fall 2018. We performed the sowing in five rounds, one per site, so that transplantation could be done at the same plant phenological stage and under similar fall conditions at the sites. Before starting, we randomly selected five seed families per population and species (or ten seed families per species when only one population was sampled). For each round of sowing, seeds were first treated with gibberellic acid (Merk KGeA, Dornstadt, Germany; 500 ppm, 500 µL per 1.5 mL microcentrifuge tube) and stored under cold conditions (dark, 4-7 °C) for one week to promote synchronous germination. Two seeds of each family were then sown in each of two individual pots of multipot-trays (diameter of 6 cm and depth of 7 cm / volume of 0.22 L, with 28 pots per tray, each perforated at the bottom, Quick-Pot QP; gvz-rossat.ch, Oetelfingen, Switzerland). Pots were filled with a mix of soil (bark compost, peat and perlite, Aussaat- und Pikiererde, Oekohum; oekohum.ch, Herrenhof, Switzerland) and sand (0-4 mm) in a ratio of 2:1 in volume. After sowing, pots were moved to growth cabinets (GroBanks; CLF, Wertingen, Germany) and kept under control conditions until transplantation (8 h of light at 18 °C, 16 h dark at 14 °C; light intensity: 200 µmol m-2 s-1; intensity was gradually increased up to 350 µmol m-2 s-1- before transplanting). Pots were thinned to one plant and some left-over plants were transferred to pots of the same family (or population) without germination. When moved to field transplant sites, plants had reached the two- or four-leaf stage (see Table S2 for dates).

Methods S3 Herbivore sampling. At each of the 5 transplant sites, sweep net sampling was performed starting 2 m away from the transplanted plants, on two orthogonal transects of 10 m length, with one sweep each meter using a sweep net of 50 cm diameter (1-mm mesh net; Bioform, Nürnberg, Germany). We performed this sampling technique twice during the growing season (July-August 2019). Furthermore, we placed five pitfall traps at each site, positioning three traps parallel to the longest side of the transect and two traps parallel to the shortest side of the blocks, with each trap equidistant (1.5 m) from the experimental blocks. Each pitfall trap consisted of a plastic container (200 mL) filled with an ethylene glycol solution (concentration of 50%, for herbivore killing and conservation), covered with a plastic roof for excluding rain. We checked the pitfall traps weekly for 4 weeks during the growing season (May-July 2019, see Table S2 for further details on dates). Sampled herbivores were collected and stored in ethanol solution (70%) for later counting and identification in the laboratory.

Methods S4 Modeling growth and extracting growth parameters. Prior to modeling growth, the mean length of the two longest leaves on the day of germination was assumed to be 0.2 cm. Furthermore, we excluded size data once size decreased by over 10% on two consecutive observations, indicating plant senescence. We also excluded individuals with fewer than five data points ($n < 5$). Then, while the three-parameter logistic model turned out to perform best across all plants, it could not be fit to all of them – to extract the growth parameters. For 17 individuals, the increase in leaf length had started slowing down by the last round of measuring; for these plants, we extrapolated the size trajectory for another three weeks. Predicted size was estimated by assuming that the increase at each time point followed the pattern of previous time points. Specifically, the increase at a given time point was estimated as the increase at the previous time point multiplied by the ratio of the previous increase to the increase from two prior time points. Still, 40 individuals remained, for which the three-parameter logistic model could not be applied. For these, the maximum mean leaf length measured was used as an estimate of asymptotic size; growth rate and time to half size received missing values (NA).
